# Mg-, Zn-, and Fe-Based Alloys With Antibacterial Properties as Orthopedic Implant Materials

**DOI:** 10.3389/fbioe.2022.888084

**Published:** 2022-05-23

**Authors:** Ning Wang, Yutong Ma, Huixin Shi, Yiping Song, Shu Guo, Shude Yang

**Affiliations:** ^1^ Department of Plastic Surgery, The First Hospital of China Medical University, Shenyang, China; ^2^ Department of Breast Surgery, The First Hospital of China Medical University, Shenyang, China; ^3^ Liaoning Provincial Key Laboratory of Oral Diseases, School of Stomatology and Department of Oral Pathology, School of Stomatology, China Medical University, Shenyang, China

**Keywords:** magnesium-based alloys, zinc-based alloys, iron-based alloys, degradable alloys, orthopedic implants, antibacterial

## Abstract

Implant-associated infection (IAI) is one of the major challenges in orthopedic surgery. The development of implants with inherent antibacterial properties is an effective strategy to resolve this issue. In recent years, biodegradable alloy materials have received considerable attention because of their superior comprehensive performance in the field of orthopedic implants. Studies on biodegradable alloy orthopedic implants with antibacterial properties have gradually increased. This review summarizes the recent advances in biodegradable magnesium- (Mg-), iron- (Fe-), and zinc- (Zn-) based alloys with antibacterial properties as orthopedic implant materials. The antibacterial mechanisms of these alloy materials are also outlined, thus providing more basis and insights on the design and application of biodegradable alloys with antibacterial properties as orthopedic implants.

## 1 Introduction

Currently, orthopedic implants have been broadly utilized to treat orthopedic and maxillofacial diseases, including deformity, osteoarthritis, and fracture (Kynaston-Pearson et al., 2013; Agarwal and García, 2015; Key et al., 2021; Farjam et al., 2022). Nevertheless, these implants are at risk of bacterial infection (Oliva et al., 2021). Actually, implant-associated infections (IAI) are among the most prevalent and severe complications in orthopedic surgery (Pfang et al., 2019). The occurrence of IAI not only means the failure of implant surgery but also requires secondary surgical repair and antibacterial therapy, which will inevitably increase the mental and economic pressure on patients (Peng et al., 2021a). IAI is primarily caused by bacteria located in the surgical approach and surgical site or brought in through blood and open wounds (Sendi et al., 2011; Tian et al., 2021). Biofilms that exert a protective effect on bacteria are formed on the surface of implants after the attachment and colonization of bacteria (Wagner and Hänsch, 2017). Clinically, antibiotic therapy remains the mainstay of treatment (Zimmerli and Sendi, 2017). Antibiotic-eluting strategies based on local diffusion are developed to address the issue of failure to achieve adequate concentrations at the site of infection by systemic use of antibiotics (Fei et al., 2011; Makarov et al., 2013). Although it is a big leap in antibacterial treatment, the ever-increasing occurrence of IAI remains unsolved. On the one hand, the formation of bacterial biofilm will be resistant to antibiotic treatment (Wagner and Hänsch, 2017). On the other hand, the release period of antibiotic-eluting devices can be short. Besides, the excessive use of antibiotics may greatly contribute to the emergence of drug-resistant bacteria (Gullberg et al., 2011). Therefore, an alternative is urgently needed to avoid the short life cycles of antibiotic-eluting devices and provide the implants with a lasting antibacterial effect. In light of this, the inherent antibacterial properties of some implant materials have gained increasing attention from researchers (Tran et al., 2015; Xu et al., 2016; Bee et al., 2021; Calabrese et al., 2021; Chen et al., 2021).

Alloy materials have always been favored in the area of orthopedic implants for their both excellent mechanical strength and mature fabrication process. There are numerous studies on traditional alloy materials, including stainless steel, cobalt-chromium (Co-Cr) alloys, and Ti alloys (Yamanaka et al., 2013; Al Jabbari, 2014; Li et al., 2014a; Bekmurzayeva et al., 2018; Xue et al., 2020). Researchers focused on enhancing the antibacterial effect of these traditional alloys during the early stage and attained fruitful results (Resnik et al., 2020; Guo et al., 2021a; Wang et al., 2021a; Lu et al., 2021; Watanabe et al., 2021). However, as permanent implants, these traditional alloy materials have many problems that need to be overcome. For instance, they carry the risk of complications such as intoxications and allergies, stress shielding problems, and secondary surgeries for implant removal (Sumner, 2015; Seyhan et al., 2018). In contrast, biodegradable alloys possess sufficient mechanical strength and can progressively degrade *in vivo*. Besides, a smaller host response ensues (Aghion, 2018). Moreover, the degradation process allows for shifting loads to healing tissues gradually, which resolves the stress shielding issues (Yuan et al., 2022). Furthermore, after full healing of tissues, the complete degradation of alloy materials obviates the need for secondary surgery (Zheng et al., 2014). Thus, biodegradable alloys have recently received significant attention, and there is a gradual increase in studies of biodegradable alloys with antibacterial properties.

At present, biodegradable alloys that are widely studied in the field of orthopedic implants include magnesium- (Mg-), iron- (Fe-), and zinc- (Zn-) based alloys. Although it is promising for the applications of biodegradable orthopedic implants with antibacterial properties, the development and application are still at an initial and exploratory stage. This review summarizes the recent advances in biodegradable Mg-, Fe-, and Zn-based alloys with antibacterial properties as orthopedic implant materials. The antibacterial mechanisms of these alloy materials are also outlined, thus providing more basis and insights on the design and application of biodegradable alloys with antibacterial properties as orthopedic implants.

## 2 The Main Pathogens and Prevention Strategies of Implant-Associated Infections

In general, the Gram-positive strains *Staphylococcus aureus* (*S. aureus*) and *Staphylococcus* epidermidis (*S. epidermidis*) are the most common causative agents of IAI in orthopedics (Arciola et al., 2005; Montanaro et al., 2011). They account for more than 70% of various causative agents (Arciola et al., 2005), followed by Gram-negative strains (*Pseudomonas* genus and Enterobacteriaceae) (Arciola et al., 2005). Depending on the site and type of implant and the timing of the infection, the cause of IAI will vary. For example, implants in the pelvis are more susceptible to be infected by Enterobacteriaceae, while *S. aureus* is still the main cause of implant surgery in other parts (Arciola et al., 2005). In addition, according to recent classification criteria, the manifestation of infections associated with orthopedic implants within 1 month after surgery is defined as early infection (Zimmerli, 2014). The virulent *S. aureus* is the main pathogen of this early perioperative infection and hematogenous infection. In most cases, chronic infections are caused by low-virulence bacteria such as coagulase-negative staphylococci (Trampuz and Widmer, 2006). No matter what kind of bacteria invade the implantation site, they will experience the process of adhesion and colonization on the implant surface, eventually persisting through the formation of stubborn biofilms (Masters et al., 2022). Clinically, systemic antibiotic therapy remains the mainstay of treatment (Zimmerli and Sendi, 2017). However, at the site of infection, antibiotics cannot reach effective concentrations (Noukrati et al., 2016). Biofilm formation often leads to the failure of antibiotic therapy (Wagner and Hänsch, 2017). At the same time, with the emergence of drug-resistant strains such as methicillin-resistant *Staphylococcus aureus* (MRSA), the treatment of IAI faces more challenges (Li and Webster, 2018).

Strategies for IAI mainly start from three aspects: 1) preventing the initial adhesion of bacteria, 2) destroying the biofilm that is just starting to form, and 3) destroying the mature biofilm. Many research studies have been devoted to improvements in antibiotic therapy, such as bone cement (Ismat et al., 2021), biopolymers (Kasza et al., 2021), ceramic materials (Cyphert et al., 2021), hydrogels (Garg et al., 2021), and nanomaterials (Keskin et al., 2021; Nag et al., 2021). They are designed as local drug delivery vehicles or coatings. This local drug delivery system successfully overcomes the problem of low blood drug concentration at the site of infection. However, there are still disadvantages, such as uneven drug release and short life cycles (Li et al., 2021). In addition, in order to solve the problem of bacterial resistance, many novel antibacterial substances, including antimicrobial peptides (Rai et al., 2022), bacteriophages (Kim et al., 2021), and nanoparticles (Nag et al., 2021), have been developed for the loading of drug delivery systems. Recently, the design of implants with antibacterial properties has begun to attract researchers’ attention. Surface modification (Narayana and Srihari, 2019; Khalid et al., 2020) and coating (Narayana and Srihari, 2019; Ahmadabadi et al., 2020) of implants are methods that have been extensively studied. These two methods are used to modulate the antibacterial properties of the implant surface. Sometimes, there are disadvantages, such as the problem of antibacterial aging. Unlike these two methods, metal alloying can achieve the overall adjustment of the implant. Antibacterial alloys can bring durable and stable antibacterial properties (Ren and Yang, 2017). At present, there are many studies on the alloying and antibacterial modification of traditional alloy materials such as stainless steel, Co-Cr alloys, and Ti alloys, and fruitful results have been achieved (Resnik et al., 2020; Guo et al., 2021a; Wang et al., 2021a; Lu et al., 2021; Watanabe et al., 2021). However, these permanently implanted alloys still suffer from unresolved drawbacks, including the risk of poisoning and allergies, stress shielding issues, and secondary surgery for implant removal (Sumner, 2015; Seyhan et al., 2018). In contrast, biodegradable alloy orthopedic implants have recently been favored by researchers due to their acceptable mechanical properties and *in vivo* degradability (Aghion, 2018; Yuan et al., 2022). Degradable Mg-, Zn-, and Fe-based alloy orthopedic implants with antibacterial properties have also been studied more, which will be described in detail below.

## 3 Research Progress of Mg-, Zn-, and Fe-Based Alloy Orthopedic Implants With Antibacterial Properties

### 3.1 Mg-Based Alloys With Antibacterial Properties

Biodegradable Mg-based alloys have been attracting much attention as orthopedic implants due to their similar mechanical properties to native bone and excellent biocompatibility (Razavi and Huang, 2019). Mg-based alloys can not only address the problem of stress-shielding related to Ti and Co-Cr alloys but also exhibit positive effects on bone regeneration (Cipriano et al., 2013; Zhang et al., 2017; Shahin et al., 2019). Additionally, Mg-based alloys can degrade naturally in the physiological condition to avoid secondary surgery to remove the implants. Thus, Mg-based alloys can be considered as a promising material for orthopedic implants (Razavi and Huang, 2019). Antibacterial properties of Mg have been gradually confirmed in recent years. A high PH environment due to degradation of Mg exhibits significant inhibition to bacteria (Robinson et al., 2010; Li et al., 2014b; Rahim et al., 2015). Nevertheless, the results of the antibacterial ability of Mg *in vivo* are not optimistic (Hou et al., 2016; Rahim et al., 2016). A reduction in antimicrobial efficacy is shown when Mg-based implants are placed *in vivo* because a high PH value is more likely to be buffered by body fluids (Bartsch et al., 2014; Zhao et al., 2020). To solve this issue, investigators are committed to adding bactericidal metal elements into Mg-based alloys in order to manufacture Mg-based alloy implants with excellent bactericidal properties.

#### 3.1.1 Antibacterial Properties

Compared with Zn- and Fe-based alloys, there are more studies on the antibacterial properties of Mg-based alloys. The research on the addition of antibacterial elements Ag and Cu is dominant. At the same time, the effects of processing methods and the addition of new antibacterial elements on the antibacterial properties of alloys are also the focus of researchers. Table 1 summarizes the antibacterial properties of existing magnesium alloys.

**TABLE 1:** Antibacterial properties of Mg-based alloys with antibacterial properties as orthopedic implants.

Alloy composition		Processing method	Antibacterial experiment	Bacterial species	Antibacterial effect	Ref.
**Mg-Ag alloys**	
	Mg-x Ag (x = 2, 4, 6 wt%)	As-cast +T4	*In vitro*	*S. aureus*	Killing rate > 90%, Mg-6Ag > Mg-4Ag > Mg-2Ag	Tie et al. (2013)
			Antibacterial tests in a bioreactor, live/dead staining, CLSM, count bacteria in solution with a nucleoCounter, and live/dead staining	*S. epidermidis*		
	Mg-x Ag (x = 6, 8 wt%)	As-cast + extrusion	*In vitro*	*S. aureus*	Killing rate > 80%, Mg-8Ag > Mg-6Ag; extruded alloy > T4 treated alloy (note: the extruded alloy has poor corrosion resistance)	Liu et al. (2017a)
		As-cast + extrusion + T4	The biofilm tests in a bioreactor, live/dead staining, CLSM, count bacteria in solution using a fluorescence microscope on a counting chamber	*S. epidermidis*		
**Mg-Cu alloys**						
	Mg-x Cu (x = 0.03, 0.19, 0.57 wt%)	As-cast	*In vitro*	*S. aureus*	After 72 h, the CFU2/ml of Mg-0.19, 0.57Cu alloy groups are almost zero, Mg-0.57Cu > Mg-0.19Cu	Liu et al. (2016)
			Plate counting method (adjust the pH of the degradation solution to neutral)			
	Mg-x Cu (x = 0.05, 0.1, 0.25 wt%)	As-cast	*In vitro*	*E. coli*	Bacterial survival, colonization, and formation of biofilm in Mg-0.1, 0.25Cu group is obviously inhibited	Li et al. (2016b)
			Spread plate method, live/dead staining, CLSM, FESEM, crystal staining to observe the formation of biofilm, qPCR analysis	*S. epidermidisMRSA*		
			*In vivoIn the MRSA-induced osteomyelitis rabbit model, radiographic analyses, histological evaluation, FESEM, microbiological evaluation*	MRSA	Mg-0.25Cu alloy can significantly inhibit bacterial invasion, alleviate inflammatory reaction, and promote the repair of bone defects secondary to infection	
	Mg-x Cu (x = 0.1, 0.2, 0.3 wt%)	As-cast + T4 (AS); As-cast + extrusion (AE); AS + AE (AES)	*In vitro* Plate counting method	*S. aureus*	After 24h, the CFU/ml of all Mg-0.1Cu alloy is almost zero, AE > AS > AES (note: the extruded alloy has poor corrosion resistance)	Yan et al. (2018a)
	Mg-x Cu (x = 0.1, 0.2, 0.4 wt%)	As-cast	*In vitro*	*P. gingivalis*	Bacterial survival and formation of biofilm in Mg-0.1Cu group is obviously inhibited	Zhao et al. (2020)
			Plate counting method, live/dead staining, SEM analysis, TEM analysis	*A. actinomycetemcomitans*		
**Mg-Zn alloys**						
	Mg-2Zn-0.5Ca (ZC21); Mg-2Zn-0.5Ca (ZSr41)	As-cast + extrusion	*In vitro* Plate counting method, SEM analysis	MRSA	ZC21 reduces bacterial adhesion more significantly than ZSr41 and Mg	Zhang et al. (2020)
	Mg-3Zn-0.5Zr (ZK30)-x Ag (x = 0.25, 0.5, 0.75, 1 wt%)	SLM	*In vitro* Plate counting method (adjust the pH of the degradation solution to neutral)	*E. coli*	The antibacterial ability of the alloy after adding Ag is obviously enhanced and proportional to the amount of Ag added	Shuai et al. (2018a)
	Mg-Zn-Y-N-x Ag (x = 0.2, 0.4, 0.6, 0.8 wt%)	As-cast + extrusion	*In vitro*	*E. coli*	The antibacterial properties of the alloys are proportional to the amount of Ag added, and Mg-Zn-YNd-0.4Ag has shown good antibacterial properties	Feng et al. (2018)
			Plate counting method	*S. aureus*		
	Mg-Nd-Zn-Zr (JDBM) alloy	As-cast	*In vitro*	*E. coli*	JDBM alloy can significantly inhibit bacterial survival, adhesion, colonization, and formation of biofilm	Qin et al. (2015a)
			Plate counting method, SEM analysis, TEM analysis	*S. aureus* *S. epidermidis*		
			*In vivo*In the MRSA-induced implant-related femur osteomyelitis model in rats, radiographic and micro-CT, histopathologic evaluation, microbiological evaluation	MRSA	JDBM alloy can inhibit bacterial invasion, alleviate inflammatory reaction, and promote new bone formation	
		SLM	*In vitro*	*E. coli*	The antibacterial ratios of the alloy are more than 90% and 3D-printed alloy can inhibit bacterial adhesion and colony formation	Xie et al. (2022)
			Determine the bacterial growth and calculate the antibacterial ratio using a microplate reader, plate counting method, live/dead staining, fluorescence microscopy	MRSA		
			*In vivo*In the MRSA-induced femur osteomyelitis rabbit model, radiographic analysis, histological evaluation	MRSA	3D-printed JDBM alloy can inhibit bacterial invasion and promote new bone formation. Immunomodulatory antibacterial properties of the alloy are confirmed	
Mg-0.1Sr, Mg- 0.1Ga, Mg-0.1Sr- 0.1Ga alloy		As-cast	*In vitro*	*E. coli*		Gao et al. (2019a)
			Plate counting method, live/dead staining, CLSM	*S. aureus* *S. epidermidis*	Ga/Sr-containing Mg-based alloys exhibit superior antibacterial properties	
			*In vivo*In the MRSA-induced femur implant-related osteomyelitis rabbit model, microbiological evaluations, histopathologic studies	MRSA	Ga/Sr-containing Mg-based alloys have good inhibitory effects on bacterial adhesion *in vivo*	
		

CLSM: confocal laser scanning microscopy; CFU: colony-forming unit; FESEM: field-emission scanning electron microscopy; SEM: scanning electron microscope; TEM: transmission electron microscopy.

Mg-Ag alloys are among the first implants studied with available antibacterial activities. As is well known, silver (Ag) has resistance to many bacterial species and was used as an essential metal fungicide very early in the past (Besinis et al., 2014). Tie et al. attempted to alloy Mg with Ag element and then manufactured three kinds of solution- (T4-) treated Mg-Ag alloys with Ag mass fractions of 1.87%, 3.82, and 6.00%, respectively. *In vitro* experiments revealed that the killing rate of three kinds of alloys on *S. aureus* and *S. epidermidis* all exceeded 90%. With the increase in silver content, the antibacterial properties of the alloys were enhanced (Tie et al., 2013). The high silver content Mg-x Ag (x = 6, 8 wt%) prepared by Liu et al. showed strong antibacterial ability in the medium containing many bacteria. However, compared with T4-treated Mg-6Ag alloy, the inhibitory effect of T4-treated Mg-8Ag alloy on bacterial viability was slightly enhanced. In addition, the as-extruded Mg-Ag alloys had stronger antibacterial properties than the T4-treated Mg-Ag alloys (Liu et al., 2017a). Unfortunately, there is no further *in vivo* translational research on Mg-Ag alloys as orthopedic implants. Several recent studies have focused on the application of Ag as an antimicrobial additive for the microalloying of other magnesium alloys, which will be mentioned below.

Mg-Cu alloys are regarded as a promising candidate for orthopedic implants because of their dual antibacterial and osteogenesis properties (Jacobs et al., 2020). Copper (Cu) is an antibacterial metal that was applied to medical treatment long ago (Szymański et al., 2012; Vincent et al., 2018). Besides, as an essential trace element in human tissue, Cu exhibits reliable physiological safety (Mitra et al., 2020). More importantly, Cu is confirmed to bring a beneficial effect on promoting osteogenesis and angiogenesis potential (Wu et al., 2013; Li et al., 2016a). This dual performance has been well applied to orthopedic implants such as Cu-doped stainless steel and Ti alloys (Ren et al., 2015; Zhao et al., 2019; Moniri Javadhesari et al., 2020; Yang et al., 2021a). Therefore, it is of great attraction for researchers to incorporate Cu into pure Mg and fabricate Mg-Cu alloys. In earlier *in vitro* studies, Mg-Cu alloys demonstrated excellent antibacterial effectiveness. The antibacterial abilities of the Mg-x Cu (x = 0.03, 0.19, 0.57 wt%) alloys prepared by Liu et al. were significantly better than those of pure Mg. With the increase in the Cu content, the antibacterial properties of the alloys were enhanced (Liu et al., 2016). Subsequently, Li et al. developed cast Mg-Cu alloys with Cu addition of 0.05, 0.1, and 0.25 wt%, respectively, in which all demonstrated broad-spectrum antimicrobial activity against *Escherichia coli* (*E. coli*), *S. epidermidis*, and MRSA and remarkably resisted bacterial adhesion and biofilm formation. Mg-0.25Cu alloy, with the best antibacterial activities and biocompatibility, was applied to a rabbit tibia model with chronic osteomyelitis. The results revealed that Mg-0.25Cu alloy could significantly inhibit the invasion of bacteria and stimulate the repair of bone defects secondary to infection (Li et al., 2016b). Regrettably, although the Mg-Cu alloys show a certain application prospect in treating osteomyelitis, they are not suitable for use as a filling material for bone defects due to their rapid degradation rate. In addition, the processing technology will affect the antibacterial effect of Mg-Cu alloys. The T4-treated Mg-0.1Cu alloy showed a delayed sterilization effect after 6 h. In contrast, the as-cast Mg-0.1Cu alloy achieved a rapid and potent killing effect on *S. aureus*, which may be attributed to higher and faster OH^−^ release than the T4-treated alloy (Yan et al., 2018a). In fact, the variability in Cu adding amounts and processing conditions enables Mg-Cu alloys to possess adjustable mechanical properties and degradation rates to adapt to diverse environments, thereby broadening the applications. Moreover, Mg-Cu alloys can not only play a role in the common causative agents of IAI, such as *S. aureus* as described above but also have a killing effect on other bacteria. In a related study, Mg–x Cu (x = 0.1, 0.2, 0.3 wt%) alloys exhibited antibacterial efficiency of up to 99.9% against *Candida* albicans (*C. albicans*) (Chen et al., 2018). Mg-Cu alloys, regarded as periodontal bone substitutes, have been used to treat periodontitis related to alveolar bone defects. It is corroborated that Mg-Cu alloys significantly decreased the survival ratios of key pathogens such as *Porphyromonas gingivalis* (*P. gingivalis*) and *Aggregatibacter actinomycetemcomitans* (*A. actinomycetemcomitans*) in periodontal diseases and peri-implantitis (Zhao et al., 2020).

Mg-Zn alloys have long been receiving substantial attention in the field of orthopedic implants because of their excellent mechanical and biomedical properties (Zhang et al., 2010; Chen et al., 2011; Seyedraoufi and Mirdamadi, 2013; Han et al., 2014; Hofstetter et al., 2015). Considered an essential element for our bodies, zinc (Zn) is safe and reliable (Zhang et al., 2021a). Moreover, Zn is verified to facilitate bone mineralization (Luo et al., 2014). However, the clinical application of Mg-Zn alloys is restricted due to rapid degradation (González et al., 2012). Several studies have put their effort into adding the third kind of alloying element for further modification of Mg-Zn alloys (Fazel Anvari-Yazdi et al., 2016; Bian et al., 2018; Prakash et al., 2018; Song et al., 2018; Miao et al., 2019). With the increasing attention to the antibacterial properties of alloys, the antimicrobial performance of some developed Mg-Zn alloys started to be explored (Zhang et al., 2020). At the same time, several novel Mg-Zn alloys with antibacterial alloying elements emerge (Shuai et al., 2018a; Feng et al., 2018). Zhang et al. evaluated the antibacterial performance of alloy pins made out of Mg-2Zn-0.5Ca (named ZC21) alloy and Mg-4Zn-1Sr (named ZSr41) alloy with excellent degradable properties and biocompatibility *in vitro*. It was revealed that ZC21 showed better antimicrobial activities than ZSr41 and pure Mg (Zhang et al., 2020). Excellent antibacterial performance is also shown in Zn. With this in mind, coupled with the antibacterial performance of zircon (Zr), both Mg-3Zn-0.5Zr (ZK30) (Shuai et al., 2018a) and Mg-6Zn-0.5Zr (ZK60) alloys (Shuai et al., 2018b) have been confirmed to have a certain antibacterial ability. Similarly, Qin et al. evaluated the antibacterial potency of Mg-Nd-Zn-Zr alloy (named JDBM) that had been developed before. The results confirmed that JDBM showed strong bacteriostatic activity against *E. coli*, *S. epidermidis*, and *S. aureus*. Moreover, JDBM appears to be a potential antibacterial orthopedic implant because of its capability of preventing infection and promoting the formation of new bones in rat models (Qin et al., 2015a). Recently, Xie et al. prepared 3D-printing JDBM implants with porous structure using selective laser melting (SLM) technology (Xie et al., 2022). The antibacterial rates of JDBM implants against *S. aureus* and *E. coli* reached 90.0% and 92.1%, respectively. Moreover, 3D-printed JDBM implants performed excellently in the rabbit femoral osteomyelitis model (Xie et al., 2022). The first attempt to apply 3D-printing technology to Mg-based alloys exhibits its potential in the field of Mg-based alloy orthopedic implants with antibacterial properties.

Adding Ag or Cu elements to the existing Mg-based alloys with superior properties has also been shown to impart or improve the antibacterial properties of the alloys. This antibacterial effect is also proportional to the amount of Ag or Cu elements added (Shuai et al., 2018a; Shuai et al., 2018b; Feng et al., 2018; Bakhsheshi-Rad et al., 2019). For example, Dai et al.’s study, which added 1 wt% Ag to Mg-4Y alloy, made the alloy’s antibacterial rate against *S. aureus* reach 92.93% (Dai et al., 2018). In Feng et al.’s, the Mg-Zn-Y-Nd-x Ag (x = 0.2, 0.4, 0.8 wt%) alloys exhibited broad-spectrum antibacterial properties against *S. aureus* and *E. coli*. The alloy already showed strong antibacterial efficacy when the Ag content reached 0.4 wt% (Feng et al., 2018). Although the ZK30 alloy already has some antibacterial properties, the addition of 0.25 to 1 wt% Ag or 0.1 to 0.3 wt% Cu will significantly improve its antibacterial ability (Shuai et al., 2018a). The addition of Cu to the ZK60 alloy was also confirmed to significantly improve the antibacterial properties of the alloy. The extracts of ZK60-0.8Cu alloy eliminated bacterial colonies within 48 h, while the extracts of ZK60-0.2Cu alloy needed 96 to achieve this effect (Shuai et al., 2018b).

The addition of electrochemically inert elements such as Ag and Cu into Mg-based alloys triggers galvanic corrosion and accelerates degradation, harming the biocompatibility and life span of Mg-based alloy implants. With this in mind, researchers tried to find new alloying elements with antibacterial properties to fabricate Mg-based alloy implants with superior corrosion resistance, antibacterial properties, and osteogenic capabilities. Along this line, Mg-based alloys containing trace content of Ga/Sr (0.1 wt%) have been developed (Gao et al., 2019a). Adding Ga/Sr shows an improvement in corrosion resistance of Mg-based alloys and displays broad-spectrum antibacterial activity against *S. aureus*, *S. epidermidis*, and *E. coli*. In addition, Mg-based alloys with Ga/Sr effectively inhibited bacterial infections in the mouse femoral osteomyelitis model (Gao et al., 2019a). The findings may shed new light on the development of antibacterial orthopedic implants. We might also shift the focus to novel, high-quality alloying elements with antibacterial properties and even osteogenic ability.

Overall, most studies on Mg-based alloy orthopedic implants with antibacterial properties are focused on classical antibacterial elements Ag and Cu. The influence of their addition on the antibacterial properties of alloys is still a subject of concern. Mg-Cu alloys are supposed to be potential orthopedic implants with double antibacterial and osteogenic effects. At the same time, it seems quite promising to further alloy Mg-Zn alloys to develop multi-element antibacterial Mg-based alloys. It is supposed to be a good idea to apply novel, high-quality alloying elements with antibacterial properties and even osteogenic ability to Mg-based alloys. Additionally, the processing technology also affects the antibacterial properties of Mg-based alloys. The application of new fabrication processes, such as 3D printing, also has expectable perspectives. However, it should be noted that the balance between antibacterial properties, mechanical properties, corrosion resistance, and biocompatibility of alloys is always an issue to be properly addressed, regardless of which way of thinking we choose.

#### 3.1.2 Mechanical Properties

During the development of antibacterial Mg-based alloys, the alloying of metal elements can not only improve their antibacterial properties but also show significant influences on their mechanical properties. Considering the “stress shielding” problem, a discussion on the mechanical properties of Mg-based alloys is warranted. The mechanical parameters of existing antimicrobial Mg-based alloys are summarized in Table 2.

**TABLE 2:** Mechanical properties of Mg-based alloys with antibacterial properties as orthopedic implants.

Alloy composition	Processing method	Vickers hardness/HV	Yield strength/MPa	Ultimate compressive strength (UCS)/MPa	Ultimate tensile strength (UTS)/MPa	Elongation	Ref.
Mg-2Ag	As-cast	32.9 ± 2.0	-	-	-	13.0	Tie et al. (2013)
Mg-4Ag		35.6 ± 1.7	-	-	-	-	
Mg-6Ag		35.9 ± 1.1	-	244.1 ± 9.2	215.9 ± 11.3	20.0	
Mg-2Ag	As-cast	5.07	-	46.09	17.26	-	Tie et al. (2014)
Mg-4Ag		7.81	-	27.17	21.32	-	
Mg-6Ag		8.157	-	25.17	21.98	-	
Mg-4Ag	As-cast	37 ± 1	30 ± 3	220 ± 12	-	-	Bryla et al. (2020)
	As-cast + homogenization + T4	41 ± 1	31 ± 2	290 ± 8	-	-	
	As-cast + homogenization + T4 + ECAP	54 ± 2	62 ± 5	325 ± 12	-	-	
Mg-1Sr-0.5Ag	Semi-solid rheo-extrusion	-	-	-	223.7	-	Tie et al. (2022)
Mg-Zn-Y-Nd	As-cast	48	-	-	-	-	Feng et al. (2018)
Mg-Zn-Y-Nd-0.2Ag		50.3	-	-	-	-	
Mg-Zn-Y-Nd-0.4Ag		50	-	-	-	-	
Mg-Zn-Y-Nd-0.6Ag		55.6	-	-	-	-	
Mg-Zn-Y-Nd-0.8Ag		51.2	-	-	-	-	
Mg-4Y-1Ag	As-cast	-	-	-	146	3.8	Dai et al. (2018)
	As-cast + extrusion	-	-	-	211	40.4	
ZK30	SLM	66.7 ± 3.5	105.3 ± 5.6	-	-	-	Shuai et al. (2018a)
ZK30-0.25Ag		78.1 ± 4.8	122.7 ± 6.5	-	-	-	
ZK30-0.5Ag		89.7 ± 4.9	134.5 ± 6.8	-	-	-	
ZK30-0.75Ag		95.5 ± 5.4	142.8 ± 7.2	-	-	-	
ZK30-1Ag		101.3 ± 6.3	130.4 ± 8.3	-	-	-	
Mg-0.1Cu	As-cast	-	58.66	-	97.3	2.8	Yan et al. (2018a)
	As-cast + T4	-	41	-	71.3	2.9	
	As-cast + extrusion	-	184.6	-	233.3	5.4	
	As-cast + extrusion + T4	-	97	-	115	3.8	
Mg-0.03Cu	As-cast	31.94	-	199.67	-	-	Liu et al. (2016)
Mg-0.19Cu		37.10	-	-	-	-	
Mg-0.57Cu		38.12	-	-	104.00	-	
Mg-1Al-0.25Cu	Ball milling + as-cast + spark plasma sintering (SPS)	-	94 ± 4	-	-	-	Safari et al. (2019)
Mg-1Al-0.5Cu		-	75.2	-	-	-	
Mg-5Zn-0.5Cu	Powder metallurgy	36.43	-	-	-	-	Purniawan et al. (2018)
Mg-5Zn-1Cu		40.13	-	-	-	-	
Mg-5Zn-1.5Cu		41.03	-	-	-	-	
ZK60	SLM	80.5 ± 1.9	131.6 ± 3.5	-	-	-	Shuai et al. (2018b)
ZK60-0.4Cu		-	158.3 ± 5.1	-	-	-	
ZK60-0.8Cu		105.2 ± 2.9	-	-	-	-	
Mg-3.16Nd-0.18Zn-0.41Zr (JDBM)	SLM + polishing + T4	-	54.80 ± 6.43	97.13 ± 7.58	-	-	Xie et al. (2022)

The addition of the Ag element exhibits a significant improvement in the mechanical properties of Mg-based alloys through grain refinement strengthening and precipitation strengthening (Tie et al., 2013; Shuai et al., 2018a; Feng et al., 2018). Ag-containing Mg binary alloys, whatever processing pathway, will result in a decrease in the average grain size of alloys as Ag content increases (Tie et al., 2013; Tie et al., 2014; Liu et al., 2017a). According to the Hall–Petch relationship, grain refinement brought by Ag enables alloys with better mechanical properties (Shuai et al., 2018a). Tie et al. confirmed this by investigating the mechanical properties of as-cast Mg2Ag, Mg4Ag, and Mg6Ag alloys (Tie et al., 2013; Tie et al., 2014). Based on such properties of Ag, several studies attempted to add a trace amount of Ag element to other Mg alloys to improve the mechanical and antimicrobial properties. In Feng et al.’s study, Mg-Zn-Y-Nd-xAg alloys (x = 0.2, 0.4, 0.6, 0.8 wt%) presented an increase in micro-hardness as Ag content increased (Feng et al., 2018) because of the addition of Ag, which leading to the grain refinement in alloys, an increase in the volume fraction of alloys in second phase, and a more scattered distribution pattern in Mg matrix (Shuai et al., 2018a; Feng et al., 2018). Nevertheless, it should be noted that when Ag is added to reach 1wt% in the Mg-3Zn-0.5Zr (ZK30) alloy, the alloy is likely to show lower compressive yield strength (CYS) due to a rougher precipitate phase and a lower binding strength on the interface between Mg matrix and precipitate phase (Shuai et al., 2018a).

Similarly, adding Cu is confirmed to have a favorable effect on the mechanical properties of Mg-based alloys. The hardness of Cu-containing Mg-based alloys significantly increases as Cu content rises. Shuai et al. established that the incorporation of Cu enabled the hardness of alloys to grow from 80.5 ± 1.9 HV of ZK60 alloy to 105.2 ± 2.9 HV of ZK60-0.8Cu alloy (Shuai et al., 2018b). Similar to the Ag element, the addition of Cu promotes the grain refinement of alloys and the formation of intermetallic phases with a higher stiffness than the Mg matrix (Shuai et al., 2018b; Xu et al., 2019). Grain refinement and uniformly distributed intermetallic phases bring high compressive strength. The compressive strength of ZK60 alloy increases to 158.3 ± 5.1 MPa after adding 0.4 wt% Cu (Shuai et al., 2018b). Moreover, due to the pinning effects by intermetallic phases along grain boundaries, more addition of Cu leads to an increase in tensile strength of Mg alloy. The ultimate tensile strength (UTS) of Mg-0.57Cu alloy is nearly twofold higher than that of pure Mg (Liu et al., 2016). It is important to remark that low supplement with Cu may not function apparently to the grain refinement of Mg-based alloys due to the low growth restriction factor value of Cu. This is corroborated in the investigation of Mg-xCu (x = 0.1, 0.2, 0.3 wt%) alloys by Yan et al., (2018a) and Chen et al., (2018). In this case, the slight improvement in the hardness of alloys by adding Cu is achieved mainly through increasing intermetallic phases (Xu et al., 2019). Nevertheless, adding too much Cu attenuates the improvement of mechanical properties. Besides the number of intermetallic phases, their size and distribution also affect the mechanical behavior (Golafshan et al., 2017). In the study of Mg-1Al-xCu alloys, Mg–1Al-0.25Cu with more uniformly distributed Al_2_Cu grains doubled the compressive and yield strength compared to Mg–1Al-0.5 Cu alloy (Safari et al., 2019). Shuai et al. also confirmed that as Cu content reached 0.6 and 0.8 wt%, excessive MgZnCu phase in ZK60-xCu alloy interconnected and formed networks along grain boundaries. This contributes to the disruption of continuity in the Mg matrix. During deformation, stress builds up at the junction of the intermetallic phase and Mg matrix, leading to a reduction in compressive strength (Shuai et al., 2018b).

The procedure of processing also affects the mechanical behavior of Mg-based alloys. It has been previously reported that solution (T4) treatment can dissolve the intermetallic phases in as-cast alloys, causing the hardness of alloys to decrease slightly (Tie et al., 2013; Yan et al., 2018a). Bryla et al. also confirmed that the high-temperature condition increased the Ag solubility in Mg during the T4 treatment, leading to the dissolution of dendritic structures in Mg-Ag alloys. However, they discovered that the solid solution strengthened elevated stiffness, compression strength, and CSF of as-cast Mg-Ag alloy after the homogeneous treatment process (Bryla et al., 2020). Extrusion treatment improves the hardness of alloys by structural refinement. During extrusion, high-temperature and high-pressure change coarse dendrites into equiaxed grains in alloys and lead to the dissolution or conversion into equiaxed grains of the second phase (Dai et al., 2018; Feng et al., 2018). More refined grains decrease stress concentration. Meanwhile, the increase in grain boundary after structural refinement impedes crack propagation, which remarkably improves the extensibility of Mg-based alloys (Yan et al., 2018a). Dai et al. showed that compared to as-cast Mg-4Y-1Ag alloy, yield stress, UTS, and elongation of extruded Mg-4Y-1Ag alloy all get improved (Dai et al., 2018). Equal-channel angular pressing (ECAP) is also an effective means of grain refinement in alloys. Bryla et al. stated that Mg–4% Ag alloy was subjected to twice ECAP treatment, and its average grain size decreased from 350 to 15 μm. The refinement significantly improves the hardness, CYS, and UCS of alloy (Bryla et al., 2020). In contrast, T6 aging treatment has a limited role in improving mechanical properties, although aging strengthening of Mg-based alloys can be realized as the precipitated phase is re-precipitated. For instance, the hardness of Mg–6% Ag after T6 treatment slightly increases from 36 HV5 to 43 HV5, while even a slight decline occurs in UCS (Tie et al., 2013).

#### 3.1.3 Corrosion Resistance

The corrosion resistance, biocompatibility, and antibacterial properties of Mg-based alloys are intimately interlinked. Mg-based alloys degrade with a concomitant elevation of pH value, osmotic pressure, and release of other metal elements. Sometimes, due to concerns about antimicrobial properties, a higher pH value and more release of ions are expected. Nonetheless, the non-negligible thing is that hyperosmolarity and excessive released ions brought by rapid degradation may result in cellular toxicity. To meet the demand for biocompatibility, the corrosion resistance of Mg-based alloys requires improvement to tightly control the degradation rate. Table 3 lists a summary of the corrosion-resistant performance of antimicrobial Mg-based alloys with various compositions and procedures of processing.

**TABLE 3:** Corrosion resistance of Mg-based alloys with antibacterial properties as orthopedic implants.

Alloy composition	Processing method	Medium/solution	Measurement	Ecorr (V)	Icorr (μA/cm^2^)	Corrosion rate (mm/year)	Ref
Mg-1.87Ag	Cast + T4	DMEM + FBS	Electrochemical analysis	-	-	0.343 ± 0.027	Tie et al. (2013)
Mg-3.82Ag						0.381 ± 0.021	
Mg-6.00 Ag						0.435 ± 0.016	
Mg-2Ag	Cast + homogenization + extrusion + drawn	DMEM + FBS	Immersion test	-	-	Pw7d = 0.473 ± 0.038	Jähn et al. (2016)
Mg-2Ag	Cast	DMEM + FBS	Electrochemical analysis	−1.42 ± 0.05	38.8 ± 0.70	0.88 ± 0.01	Tie et al. (2014)
Mg-4Ag				−1.41 ± 0.03	51.8 ± 3.03	1.01 ± 0.03	
Mg-6Ag				−1.38 ± 0.05	53.4 ± 1.10	1.18 ± 0.02	
Mg-6Ag	Cast + homogenization + extrusion	CCM, DMEM, GlutaMAX + FBS	Immersion test	-	-	Pw7d < 0.5	Liu et al. (2017a)
Mg-8Ag				-	-	Pw7d = 3.47	
Mg-8Ag	Cast + homogenization + extrusion + T4			-	-	Pw7d < 0.5	
ZK30	SLM	SBF	Electrochemical analysis	−1.64 ± 0.04	109.6 ± 4.5	2.39 ± 0.22	Shuai et al. (2018a)
ZK30–0.25Ag				−1.52 ± 0.05	81.1 ± 4.2	1.77 ± 0.15	
ZK30-0.5Ag				−1.53 ± 0.03	64.5 ± 4.5	1.41 ± 0.13	
ZK30–0.75Ag				−1.54 ± 0.02	74.3 ± 3.5	1.62 ± 0.16	
ZK30-1Ag				−1.56 ± 0.03	120.23 ± 6.7	2.62 ± 0.25	
Mg-4Y-1Ag	Cast	DMEM + FBS	Immersion test	-	-	Pw7d < 0.5	Vlcek et al. (2017)
	Cast + T4			-	-	Pw7d < 0.3	
Mg-4Y-1Ag	Cast	PBS	Electrochemical analysis	−1.537	13.2	-	Dai et al. (2018)
	Cast + homogenization + extrusion			−1.464	5.38	-	
Mg-x Cu (x = 0,0.05,0.1,0.25 wt%)	Cast	Hank’s	Immersion test	-	-	Increased with increasing Cu content	Li et al. (2016b)
Mg-0.1Cu	Cast	0.9 wt% NaCl				Pw24h ≈ 25	Chen et al. (2018)
Mg-0.3Cu			Immersion test	-	-	Pw24h ≈ 200	
Mg-0.1Cu			Electrochemical analysis	−1·45	120	2.74	
Mg-0.2Cu				−1·47	170	3.88	
Mg-0.3Cu				−1·49	1,280	29.35	
Mg-0.1Cu	Cast	Hanks’	Immersion test	-	-	Pw7d = 49.5	Yan et al. (2018a)
	Cast + T4			-	-	Pw7d = 5.76	
	Cast + extrusion			-	-	Pw7d = 0.92	
	Cast + extrsion + T4			-	-	Pw7d = 1.7	
	Cast		Electrochemical analysis	−1.54 ± 0.02	17.40 ± 3.13	-	
	Cast + T4			−1.56 ± 0.02	3.96 ± 0.73	-	
	Cast + extrusion			−1.52 ± 0.01	8.42 ± 0.87	-	
	Cast + extrsion + T4			−1.52 ± 0.01	6.42 ± 0.89	-	
Mg-0.06Cu	Cast	Hanks’	Immersion test	-	-	Pw14d = 25 ± 1	Yan et al. (2019)
	Cast + T4			-	-	Pw14d = 0.52 ± 0.09	
ZK60	SLM	SBF	Electrochemical analysis	−1.621	44.20	1.01	Shuai et al. (2018b)
ZK60-0.2Cu				−1.584	60.39	<5	
ZK60-0.4Cu				−1.577	85.34	<5	
ZK60-0.6Cu				−1.570	48.57	>10	
ZK60-0.8Cu				−1.5	82.75	>15	
ZK30	SLM	SBF	Immersion test	-	-	Pw14d = 0.90 ± 0.02	Xu et al. (2019)
ZK30-0.1Cu				-	-	Pw14d = 0.97 ± 0.04	
ZK30-0.2Cu				-	-	Pw14d = 1.11 ± 0.02	
ZK30-0.3Cu				-	-	Pw14d = 1.32 ± 0.06	
ZK30			Electrochemical analysis	17.8	-	0.41	
ZK30-0.1Cu				28.2	-	0.64	
ZK30-0.2Cu				38.0	-	0.87	
ZK30-0.3Cu				47.8	-	1.09	
Mg-0.25Sr	Cast + homogenization treatment + extrusion	Hanks’	Electrochemical analysis	−1.81 ± 0.02	3.03 ± 0.73	0.07 ± 0.02	Liu et al. (2014)
Mg-1.0Sr				1.79 ± 0.001	2.93 ± 0.27	0.07 ± 0.01	
Mg-1.5Sr				−1.77 ± 0.02	2.93 ± 0.58	0.07 ± 0.01	
Mg-2.5Sr				−1.68 ± 0.04	1.36 ± 0.41	0.03 ± 0.01	
Mg-0.1Sr	Cast	Trypticase Soy Broth	Immersion test	-	-	Pw3d = 0.9	Gao et al. (2019a)
Mg-0.1Ga				-	-	Pw3d = 1.1	
Mg-0.1Sr-0.1Ga				-	-	Pw3d = 1.3	

CCM: cell culture medium, DMEM: Dulbecco’s Modified Eagle’s Medium, FBS: fetal bovine serum, SBF: simulate body fluids.

The addition of Ag and Cu elements increases the antimicrobial behavior, with a concomitant decrease in corrosion resistance. In Ag-containing binary Mg alloys, the occurrence of corrosion is more prone to exist as Ag content increases. Tie et al. found that Mg-x Ag (x = 2, 4, 6 wt%) alloys degraded more rapidly with the increase in Ag content, whether or not hot treatment was performed (Tie et al., 2013). Liu Z et al. suggested that the degradation rate of alloy reached 3.47 mm/year as the concentration of Ag increased up to 8.51 wt%, compared with that of pure Mg (0.5 mm/year) (Liu et al., 2017a). Cu-containing binary Mg alloys also demonstrate the same tendency (Li et al., 2016b; Liu et al., 2016; Yan et al., 2018a; Chen et al., 2018). For example, the corrosion rate of Mg-0.1Cu alloy manufactured by Li et al. is double that of pure Mg. Besides, the corrosion rate increases from 25 mm/y to nearly 200 mm/y, with Cu content growing from 0.1 to 0.3 wt% (Li et al., 2016b). The dominant reason for this phenomenon lies in the solubility of Ag and Cu in Mg. More secondary phases precipitate in alloy with increasing the Ag or Cu content (Tie et al., 2013; Li et al., 2016b; Liu et al., 2016; Liu et al., 2017a). There is a variation in electrochemical potentials between inert Mg-Ag or Mg-Cu particles and Mg matrix in these alloys (Li et al., 2016b; Bryla et al., 2020). Bulk secondary phases act as highly active micro-cathodes, coupled with α-Mg anodes, thus forming numerous micro corrosion cells leading to the accelerated corrosion of the Mg matrix (Li et al., 2016b; Liu et al., 2017a; Bryla et al., 2020). Furthermore, pitting corrosion occurs due to the different corrosion rates between the α-Mg phase and secondary phase, resulting in accelerating the non-uniform degradation process of alloy. In contrast, a slight addition of Ag or Cu element in other Mg-based alloys results in different outcomes. In the study of Shuai et al., the corrosion rate of ZK30-xAg (x = 0, 0.25, 0.5, 0.75, 1 wt%) alloy increased initially and then diminished with increased Ag content. When the Ag content reached 1 wt%, the corrosion resistance of alloy tended to be adversely affected (Shuai et al., 2018a). Zhang et al. stated that the modified Mg-based alloys were obtained by adding Ag into Mg-3.0Nd-0.2Zn-0.4Zr (named JDBM) alloys. The degradation rate of the alloys substantially accelerated with 0.4 wt% loading of the Ag content (Zhang et al., 2013). Similar results were obtained when adding the Cu element into Mg-based alloys. The corrosion resistance improves as the Mg-1Al alloy is modified by adding 0.025 wt%Cu. In contrast, the degradation rate is significantly accelerated as the Cu content reaches 0.1 wt% (Safari et al., 2019) because, on the one hand, the addition of Cu and Ag elements results in structural refinement and increases the density of grain boundaries. Fine-grain size is favorable for the formation of dense corrosion product film, while the high-density distribution of grain boundaries acts as a corrosion barrier to suppress the corrosion process (Zhang et al., 2013; Shuai et al., 2018a; Shuai et al., 2018b). On the other hand, when the addition of Ag or Cu elements reaches a certain content, more precipitate phases occur, and alloys are more likely to degrade rapidly because of the exacerbation of galvanic and pitting corrosion (Zhang et al., 2013; Shuai et al., 2018b; Feng et al., 2018; Safari et al., 2019).

As a commonly used alloying element in Mg-based alloys, Zn is corroborated to elevate corrosion resistance significantly through effective grain refinement in antibacterial Mg-based alloys (He et al., 2015; Zhang et al., 2020). Mg-2Zn-0.5Ca (named ZC21) alloys and Mg-4Zn-1Sr (named ZSr41) alloys present excellent corrosion resistance that outperform pure Mg (Zhang et al., 2020). Because of adding Zn, Nn, and Zr, the corrosion rate of JDBM alloys was even reduced fivefold compared with pure Mg (Qin et al., 2015a). After Qin et al. added 2∼4 wt% Zn into the Mg-1Ca-0.5Sr alloy, the corrosion resistance of the alloy got improved. More uniform corrosion appeared on the surface of Zn-containing alloys, with no apparent corrosion pits. Nevertheless, Mg-1Ca-0.5Sr-6Zn alloy implied a great hydrogen evolution rate (He et al., 2015). This is possibly explained by the reduction in Zn solubility in the Mg matrix due to the appearance of other alloying elements. Excessive Zn promotes the precipitation of Zn-containing intermetallic phases, thereby accelerating galvanic erosion (He et al., 2015). Moreover, the improvement of corrosion resistance in Mg-based alloys can also be observed with the addition of biocompatible elements such as Sr and Ga. However, due to the low solubility of Sr in the Mg matrix, the corrosion resistance of the Mg-Sr alloy declined due to more precipitation phases when the Sr content reached over 1wt% (Liu et al., 2014). Thus, Gao et al. added 0.1 wt% Sr and/or Ga for microalloying treatment on Mg-based alloys. The obtained Mg-0.1Sr, Mg-0.1Ga, and Mg-0.1Sr-0.1Ga showed much slower corrosion rates than pure Mg (Gao et al., 2019a).

Meanwhile, the processing procedure has a great impact on corrosion resistance. T4 treatment effectively promotes the performance of corrosion resistance. Yan et al. stated that the corrosion rate of the Mg-0.1Cu alloy (0.92 mm/y) *via* solution processing was reduced over 50-fold compared with that of the as-cast Mg-0.1Cu alloy (49.5 mm/y) (Yan et al., 2018a). T4 treatment can dissolve most of the secondary dendrites or precipitate phase, causing the surface corrosion potential to distribute more evenly and a significant reduction in the number of micro-galvanic cells (Liu et al., 2017a; Vlcek et al., 2017; Yan et al., 2018a; Feng et al., 2018; Yan et al., 2019). Meanwhile, the dissolution of precipitated particles and homogenization of solute bring a more homogenous, compact, and flat degradation surface and a lower trend toward pitting corrosion (Tie et al., 2013; Liu et al., 2017a). It is not difficult to understand that the Mg alloys exhibit more excellent corrosion resistance and degradation behavior after T4 treatment. Hot extrusion can also improve the corrosion resistance of alloys for refining grain structures and distributing intermetallic phases evenly (Yan et al., 2018a). However, there are still many intermetallic phases in alloys after extrusion. The intermetallic phases spreading along crush-bands and Mg matrix form a large cathode-to-anode area, leading to severe corrosion. Thus, the improvement of extrusion on corrosion resistance of Mg-based alloys is not obvious (Yan et al., 2018a; Yan et al., 2018b).

There is another issue that cannot be overlooked for the corrosion resistance of Mg-based alloys. Many studies have established that a clear difference is observed between *in vivo* and *in vitro* degradation of Mg-based alloys (Kumar and Katyal, 2021). Multiple factors in the *in vivo* environment may impact the degradation of Mg-based alloys. The studies on *in vivo* degradation in terms of antibacterial Mg-based alloys is of great importance (Kumar and Katyal, 2022). Jahn et al. studied the intramedullary Mg2Ag nails, finding it took 617 days for *in vitro* degradation, while only 210 days for the *in vivo* test. Although there is a certain difference in degradation between *in vivo* and *in vitro*, the *in vivo* degradation rate of Mg2Ag nails was still acceptable (Jähn et al., 2016). The shape of the ZC21 alloy was still largely maintained after 12 weeks of implantation on mouse femurs (Zhang et al., 2020). The degradation rate of JDBM in rat femur was merely 0.092 mm/y (Qin et al., 2015a). The studies on *in vivo* degradation of antibacterial Mg-based alloys are so far limited, and additional research is needed.

#### 3.1.4 Biocompatibility

As discussed above, the addition of alloying elements indeed improved the antimicrobial properties of Mg-based alloys. Nevertheless, the issue of cytotoxic effects caused by changing the pH values and releasing the metal ions has always been a concern. It is reported that most antibacterial Mg alloys present good *in vitro* biocompatibility. Table 4 summarizes the results of studies on *in vitro* biocompatibility of various antibacterial Mg-based alloys.

**TABLE 4:** *In vitro* biocompatibility of Mg-based alloys with antibacterial properties as orthopedic implants.

Alloy composition	Processing method	Measurement	Cell line	Result	Ref.
Mg-x Ag (x = 1.87,3.82,6.00 wt%)	As-cast + T4	Live/dead staining and adhesion test (14 d)	Human primary osteoblasts	Cell viability: between 95% and nearly 100%. Cells directly adhered to the alloy surface. Mg-2Ag showed the highest cell viability, nearly 100%	Tie et al. (2013)
Mg-6Ag	As-cast + homogenization + extrusion	MTT	Human primary osteoblasts	100% extract: <75% (3 d)	Liu et al. (2017a)
				20%, 10% extract: around 100% (3 d)	
Mg-8Ag	As-cast + homogenization + extrusion			100%, 20% extract: <75% (3 d)	
				10% extract: around 100% (3 d)	
Mg-8Ag	As-cast + homogenization + extrusion + T4			100% extract: <75% (3 d)	
				20%, 10% extract: around 100% (3 d)	
Mg-2Ag	As-cast + homogenization + extrusion + drawn	ALP	Mice long bone osteoblasts	Culture for 7 days with 3.3%, 10%, and 20% media conditioned with degradation products had no effect on ALP activity	Jähn et al. (2016)
		TRAP activity	Mice bone marrow-derived osteoclast precursors	The number and size of TRAP-positive multinucleated osteoclasts decreased with the increase in the concentration of degradants in the medium	
ZK30-x Ag (x = 0, 0.25, 0.5, 0.75, 1 wt%)	SLM	CCK-8	MG 63	100% extract: 64.2%–75.3% (1 d)	Shuai et al. (2018a)
				50% extract: 75.2%–81.3% (1 d)	
				ZK30-0.5Ag showed increased cell viability during culture with the best biocompatibility	
Mg-4Y-1Ag	As-cast	MTT	Mouse fibroblast L929	The relative cell growth rate (RGR) was between 83.0% and 99.4% at different dilutions of the extract and at different time points	Dai et al. (2018)
	As-cast + 400 °C/24 h+ extrusion				
Mg-1Ca-1Mn-6Zn-x Ag (x = 0, 0.5, 1, 2 wt%)	Space holder	MTT	MG 63	The 2.0 wt% Ag content alloy induced a cytotoxic response, while the other alloys caused only a slight decrease in cell viability	Bakhsheshi-Rad et al. (2019)
		ALP		APL activity decreased with increasing silver content	
Mg-x Cu (x = 0, 0.05, 0.1, and 0.25 wt%)	As-cast	CCK-8	Balb/c 3T3	The cell viability of all alloys showed a similar increasing trend, and there was no significant difference with the control Ti alloy group	Li et al. (2016b)
		Live/dead staining (2 d)		Cells grew well in all groups with long stress fiber bundles composed of actin filaments and good cell-to-cell contacts	
Mg-0.03Cu	As-cast	MTT (1.25 cm^2^/ml extraction ratio)	MC3T3-E1	>100% (1 d)	Liu et al. (2016)
Mg-0.19Cu				>100% (1 d) nearly 100% (1 d)	
Mg-0.57Cu					
Mg-0.03Cu			HUVECs	125% (1 d)	
Mg-0.19Cu				>100% (1 d)	
Mg-0.57Cu				75% (1 d)	
Mg-0.03Cu		ALP	MC3T3-E1	Increased	
Mg-0.19Cu				Increased	
Mg-0.57Cu				Decreased	
Mg-0.1Cu	As-cast	MTT	rBMSCs	100% extract: >90% (1 d), >80% (2.3 d)	Yan et al. (2018a)
	As-cast + T4			100% extract: >100% (1 d), >90% (2.3 d)	
	As-cast + extrusion			100% extract: >100% (1.2 d); >80% (3 d)	
	As-cast + extrusion + T4			100% extract: >100% (1, 2, 3 d)	
ZK60-x Cu (x = 0, 0.2, 0.4, 0.6, 0.8 wt%)	SLM	CCK-8	MG 63	Approximate to 100% (1 d)	Shuai et al. (2018b)
				79%–95% (5 d)	
				ZK60-6Cu and ZK60-8Cu exhibit relatively poor cell viability	
Mg-1Al	SPS	MTT	MG 63	Nearly 100% (1, 3 d)	Safari et al. (2019)
Mg-1Al-0.25Cu				>100% (1, 3 d)	
Mg-1Al-0.5Cu				Nearly 100% (1 d); >100% (3 d)	
Mg-1Al-1Cu				<75% (1, 3 d)	
Mg-Nd-Zn-Zr (JDBM)	Semi-continuous As-casting	Live/dead staining and adhesion test (24 h)	hBMSCs	Reduced number of viable cells and poor cell spreading on JDBM samples compared to Ti	Qin et al. (2015a)
Mg-3.24Nd-0.21Zn- 0.44Zr (JDBM)	SLM + electrochemical polishing + T4	CCK-8	MC3T3-E1	50%, 25%, 12.5% extract	Xie et al. (2022)
				>100% (1, 3, 7 d)	
			RAW 264.7	50%, 25%, 12.5% extract	
				Around 100% (1, 3, 7 d)	
		ALP	MC3T3-E1	25%, 12.5% extract	
				Increased	
Mg-1Ca-0.5Sr	As-cast + T4 + extrusion	MTT	MC3T3-E1	Nearly 100% (2 d), >100% (4 d), nearly 80% (6 d)	He et al. (2015)
Mg-1Ca-0.5Sr-2Zn				>100% (2, 4, 6 d)	
Mg-1Ca-0.5Sr-4Zn				Nearly 100% (2.4 d), >90% (6 d)	
Mg-1Ca-0.5Sr-6Zn				Nearly 100% (2.4 d), >80% (6 d)	
Mg-5.6Zn	As-cast	CCK-8	rBMSCs	Nearly 100% (1, 3 d), >100% (5 d)	Yu et al. (2016)
		ALP		Increased	
Mg-0.25Sr	As-cast + homogenization treatment + extrusion	MTT	MC3T3-E1	>90% (1 d)	Liu et al. (2014)
Mg-1.0Sr				>100% (1 d)	
Mg-1.5Sr				>100% (1 d)	
Mg-2.5Sr				>100% (1 d)	

Adding moderate Ag and Cu content does not seem to have influenced the biocompatibility of alloys. Antimicrobial Mg alloys containing Ag or Cu show no cytotoxic effects on human primary osteoblasts (Tie et al., 2013; Liu et al., 2017a), mice long bone osteoblasts (Jähn et al., 2016), MG 63 cells (Shuai et al., 2018a; Shuai et al., 2018b; Bakhsheshi-Rad et al., 2019; Safari et al., 2019), mouse fibroblast L929 cells (Dai et al., 2018), Balb/c 3T3 cells (Li et al., 2016b), MC3T3-E1 cells (Liu et al., 2016), HUVECs (Liu et al., 2016), and rBMSCs (Yan et al., 2018a). The addition of low Cu content even induces the osteogenic differentiation of osteogenic precursor cells, mineralization of extracellular matrix, and collagen secretion (Liu et al., 2016). Cu in low concentration is also conducive to enhancing the activity, proliferation, migration, and angiogenesis-related markers expression of HUVECs (Liu et al., 2016). Mg-Cu alloys under T4 treatment also present good biocompatibility because of the optimized performance of corrosion resistance (Yan et al., 2018a). Nonetheless, continued attention is required that excessive addition of Ag and Cu may adversely affect the survival, proliferation, and adhesion of cells, especially for Cu-containing Mg alloys, as confirmed by several studies (Liu et al., 2016; Shuai et al., 2018a; Yan et al., 2018a; Shuai et al., 2018b; Bakhsheshi-Rad et al., 2019; Safari et al., 2019). It is essential to consider biocompatibility, antibacterial properties, mechanical behavior, and corrosion resistance when probing the optimal addition amount of Ag and Cu in different Mg-based alloys.

The addition of biocompatible elements, such as Zn, Ca, and Sr, has been demonstrated to enhance the biocompatibility of antimicrobial Mg-based alloys. Compared with Mg-1Ca-0.5Sr, alloys with 2∼6 wt%Zn exhibit higher biocompatibility (He et al., 2015). Zhang C et al. pinyed out that because of the addition of Zn and Ca, the ZC21 alloy presented a stronger stimulatory effect on the adhesion and proliferation of BMSCs than the Ti alloy (Zhang et al., 2020). Adding Zn to Mg-Zn binary alloys is also confirmed to promote osteogenic differences in rBMSC and extracellular matrix calcium deposition (Yu et al., 2016). Furthermore, the Sr element is confirmed to contribute positively to the survival rate of hMSCs, which may counterbalance the potential adverse effects of over-releasing Mg ions (Gao et al., 2019a). Mg-Sr alloys exhibit an obvious positive promotion in the survival, proliferation, adhesion, and spreading of MC3T3-E1cells (Liu et al., 2014).

There are few studies on the *in vivo* biocompatibility of antimicrobial Mg-based alloys. Table 5 summarizes the results of studies on *in vivo* biocompatibility of several antibacterial Mg-based alloys. Additional *in vivo* researches are indispensable for better clinical translation.

**TABLE 5:** *In vivo* biocompatibility of Mg-based alloys with antibacterial properties as orthopedic implants.

Alloy Composition	Processing method	Animal model	Implantation position	Result	Ref.
Mg-2Ag pin	Cast + homogenization + extrusion + drawn	C57Bl/6J mice	Right femoral shaft simulating an open fracture	No acute or long-term systemic side effects for 131 days with good stabilization of the fracture site and bone regeneration	Jähn et al. (2016)
Mg-0.25Cu	Cast	New Zealand White rabbits	Left tibia simulating the osteomyelitis model	No systemic inflammatory response, tissue, and organ damage	Li et al. (2016b)
Mg-Nd-Zn-Zr (JDBM)	Semi-continuous casting	SD rats	Left femur simulating the osteomyelitis model	No obvious adverse reactions; the antibacterial and bone regeneration effects were good	Qin et al. (2015a)
Mg-3.24Nd-0.21Zn-0.44Zr (JDBM)	SLM + electrochemical polishing + T4	New Zealand white rabbits	Right femur simulating the osteomyelitis model	No systemic tissue and organ damage	Xie et al. (2022)
Mg-5.6Zn	Cast	SD rats	Distal femur simulating the osteomyelitis model	No obvious adverse reactions; the antibacterial and bone regeneration effects were good	Yu et al. (2016)
Mg-0.1Sr, Mg-0.1Ga, Mg-0.1Sr-0.1Ga	Cast	SD rats	Femur simulating the osteomyelitis model	No obvious adverse reactions; the antibacterial and bone regeneration effects were good	Gao et al. (2019a)

### 3.2 Fe-Based Alloys With Antibacterial Properties

Fe-based alloys are highly valuable in the field of orthopedic implants because of their excellent biocompatibility, degradability, and mechanical properties (Gorejová et al., 2019). Higher mechanical strength of Fe, compared with Mg and Zn, is essential for orthopedic implants that require shearing enough stress and loads (Heiden and Walker, 2015). Nevertheless, unlike Mg-based alloys that degrade rapidly, the slow degradation reactivity of Fe alloys restricts its clinical application (Chen et al., 2020). The degradation rate can be improved by adding alloying elements that form galvanic corrosion (Schinhammer et al., 2010; Liu and Zheng, 2011; Heiden and Walker, 2015). This is also required for the preparation of antibacterial Fe-based alloys. Unfortunately, there are relatively few studies on Fe-based alloys with antibacterial properties.

#### 3.2.1 Antibacterial Properties

Some existing studies on Fe-based alloys with antibacterial properties mainly focus on the addition of the antibacterial elements, such as Cu and Ag. Table 6 summarizes the antibacterial properties of existing Fe-based alloys.

**TABLE 6:** Antibacterial properties of Fe- and Zn-based alloys with antibacterial properties as orthopedic implants.

Alloy composition		Processing method	Antibacterial experiment	Bacterial species	Antibacterial effect	Ref.
**Fe-based alloys**	
	Fe-x Cu (x = 1.5, 2.3, 7.8, 10.1 wt%)	SLM	*In vitro*	*E. coli*	SLMed Fe-xCu alloys show strong antibacterial ability, Fe-10.1Cu > Fe-7.8Cu > Fe-2.3Cu > Fe-1.5Cu (note: degradation rate of Fe-10.1Cu alloy is too slow, and Fe-7.5Cu alloy has the best degradation rate)	Guo et al. (2021b)
			Bacterial counting method			
	Fe-8Cu alloy	Microwave sintering	*In vitro*	*E. coli*	The antibacterial rates of microwave-sintered Fe-8Cu alloy against *E. coli* are up to 99.9%	Deng et al. (2021)
			Bacterial counting method			
**Zn-based alloys**						
**Zn**-**Ag alloys**						
		As-cast	*In vitro*	*S. gordonii*	Zn-4Ag can significantly inhibit bacterial survival, adhesion, and biofilm formation	Li et al. (2018a)
			Crystal violet staining assay, fluorescent nucleic acid stain, live/dead staining, fluorescence microscope			
	Zn-0.5, 1, 2Ag	Extrusion	*In vitro*	*S. aureus*	Zn-1, 2Ag alloys show significant inhibition of bacterial survival and adhesion. Zn-2Ag has the strongest antibacterial ability	Qu et al. (2021)
			Coated plate method, FESEM, SEM, live/dead bacteria staining, CLSM, TEM, qPCR	*S. epidermidis*MRSAMRSE		
			*In vivo*In the MRSA-induced femur osteomyelitis rat model, X-ray observation, bacterial detection around implants, histomorphometric analysis	MRSA	Zn-2Ag alloy has excellent antibacterial ability *in vivo* and can inhibit the inflammatory response	
	Zn-2Ag-1.8Au-0.2V	As-cast + hot rolled	*In vitro* Live/dead staining, fluorescence microscope.	S. gordonii	Alloy shows significant inhibition of bacterial colonization and biofilm formation	Li et al. (2019c)
	Zn-1Ag-0.05Zr	Solution treatment + extrusion	*In vitro* Inhibition zone diameter (IZD) method (plate culture, take endpoint pictures using camera measure inhibition zone).	*E. coli*, *S. aureus*	Zn-1Ag-0.05Zr alloy has good antibacterial properties but is weaker than Zn-1Ag alloy (note: Ternary alloy has better mechanical strength)	Wątroba et al. (2019)
**Zn**-**Cu alloys**						
	Zn-x Cu (x = 0.5, 1, 2 wt%)	Extrusion	*In vitro*	*S. aureus*	Zn-1, 2Cu alloys show significant inhibition of bacterial survival, adhesion, and biogenesis. Zn-2Cu has the strongest antibacterial ability	Qu et al. (2020)
			Measure bacteriostatic efficiency using a serial dilution plating method, live/dead stain, CLSM, SEM, FESEM, TEM, real-time PCR	*S. epidermidis*MRSAMRSE			
			*In vivo*In the rat femur intramedullary nail infection prevention model, X-ray observation, histology, culture of bacteria from implants and surrounding tissue	MRSA	Zn-2Cu alloy shows significant antibacterial activity and alleviates inflammatory toxicity and infection-related bone loss	
	Zn-1,2,4Cu alloy	Hot rolling	*In vitro*	Mixed oral bacteria	Alloys inhibit biofilm formation	Li et al. (2019e)
			Live/dead staining, fluorescence microscope			
	Zn-1,2,3,4Cu alloy	Laser powder bed fusion	*In vitro* Agar disk diffusion method	*Escherichia coli*	Zn-Cu alloy exhibited a greatly enhanced antibacterial activity	Shuai et al. (2020)
	Zn-1Cu-0.1Ti	As-cast	*In vitro* Inhibition zone diameter (IZD) method	*S. aureus*	Alloy shows good antibacterial properties	Lin et al. (2020)
	Zn-0.5Cu-x Fe (x = 0.1, 0.2, 0.4 wt%)	Hot extrusion	*In vitro*	*S. gordonii*, mixed oral bacteria	Zn-0.5Cu-0.2Fe alloy with relatively good mechanical and corrosion properties exhibits good antibacterial properties	Zhang et al. (2021c)
			Live/dead staining, fluorescence microscope, calculate the antibacterial ratio using a microplate reader			
	Zn-11.16Cu	Electrochemical deposition	*In vitro* Inhibition zone diameter (IZD) method	*S. aureus*	Zn-Cu alloy foam shows good antibacterial effects	Tong et al. (2020)
Zn-0.5Al-x Mg (x = 0.1, 0.3, 0.5 wt%)		As-cast	*In vitro*Disc diffusion antibiotic sensitivity testing	*E. coli*, *S. aureus*	The addition of Mg improves the antibacterial ability of the alloy. Zn-0.5Al-0.5 Mg alloy > Zn-0.5Al-0.3 Mg alloy > Zn-0.5Al-0.1 Mg alloy	Bakhsheshi-Rad et al. (2017)

Zn-0.05 Mg		Hot extrusion	*In vitro* Culture of bacteria from implants and surrounding tissue	*E. coli*, *S. aureus*	The alloy exhibits strong antibacterial activity	Xiao et al. (2018)
Zn-0.8Mg-0.2Sr		As-cast + homogeniz-ation + annealing + extrusion	*In vitro*	*S. gordonii*	The alloy exhibits inhibitory effects on the adhesion and biofilm formation of *S. gordonii*	Čapek et al. (2021)
			Bacterial adhesion test (live/dead staining, fluorescence microscopy)			
Zn-0.04Mg-2Ag porous scaffold		Template replication technique	*In vitro*	*E. coli*	The alloy had little antibacterial effect on *E. coli* but had obvious antibacterial ability against *S. aureus* and *S. epidermidis*	Wu et al. (2021)
			Culture plate method	*S. aureus*, *S. epidermidi*s		
Zn-0.8Mn		Hot treatment	*In vitro*	*E. coli*	Good antibacterial ability of the alloy is insensitive to the heat treatment	Sun et al. (2020)
			Spread plate method			
Zn-0.8Mn-0.4x (x = Ag, Cu, or Ca) alloys		Hot extrusion	*In vitro*	*E. coli*	The addition of Cu or Ag endows Zn-0.8Mn alloy’s antibacterial activity against *E. coli*	Shi et al. (2019)
			Count active bacteria			
Zn-1,2,3Ce		Laser additive manufacturing technique	*In vitro* Inhibition zone diameter (IZD) method	*E. coli*	Zn-Ce exhibited good antibacterial efficiency with a bacterial inhibition rate of 81.36%	Yang et al. (2021b)

The addition of Cu element was confirmed to impart antibacterial properties to Fe-based alloys. Guo et al. suggested that Fe-x Cu (x = 0, 1.5, 2.3, 7.8, and 10.1 wt%) alloys prepared by SLM exhibited superior antibacterial properties. The antibacterial ability of the alloys was enhanced with the increase in the Cu content. The antibacterial rate of the SLMed Fe-1.5 Cu alloy against *E. coli* was about 96.5% (Guo et al., 2021b). The antibacterial rates of other high-content Fe-Cu alloys were all greater than 99.9%. Deng et al. also confirmed the antibacterial efficacy of Cu-containing Fe-based alloys. They used microwave sintering to prepare porous Fe-8Cu alloy with an antibacterial rate of 99.9% against *E. coli* (Deng et al., 2021). In addition, the excellent antibacterial properties of Cu-containing Fe-Mn alloys are unanimously affirmed. Although Fe-Mn alloys have a certain antibacterial effect or promote bacterial growth, there is some controversy (Sotoudehbagha et al., 2018; Mandal et al., 2019; Mandal et al., 2021). Mandal et al. stated that the Fe-Mn-0.9Cu alloy did not have antibacterial properties. However, when the Cu addition amount was further increased (5 and 10 wt%), the Fe-Mn-Cu alloy exhibited obvious bacterial growth inhibition with the increase of Cu content (Mandal et al., 2021). Similarly, Fe-(35-x) Mn-x Cu (x = 0, 1, 3, 5, 10 wt%) also showed an enhanced bactericidal effect on *E. coli* with the increase in copper content (Mandal et al., 2019).

However, little research has been done on Ag-doped Fe-based alloys. Sotoudehbagha et al. confirmed that when 1 wt% Ag was added to the Fe-30Mn alloy, the antibacterial rate of the alloy against *E. coli* and *S. aureus* rose to 77% and 90%, respectively. When the silver content reached 3wt%, the antibacterial rate of the alloy against *E. coli* and *S. aureus* could reach 99%.

In conclusion, although the studies on the antibacterial properties of Fe-based alloys are relatively scarce, the existing results are promising. Fe-based alloys with antibacterial properties deserve in-depth research.

#### 3.2.2 Mechanical Properties

The addition of Ag and Cu elements in the antibacterial Fe-based alloys not only gives the Fe-based alloys antibacterial properties but also improves the mechanical properties of the alloys. Table 7 summarizes the mechanical properties of antibacterial Fe-based alloys containing Cu or Ag and highlights the effects of the amount of Ag or Cu added on the mechanical properties of antibacterial Fe-base alloys.

**TABLE 7:** Mechanical properties of Fe-based alloys with antibacterial properties as orthopedic implants.

Alloy composition	Processing method	Density (g/cm^3^)	Relative density (%)	Hardness (HV)	Ref.
Fe-2Ag	Sintering	7.807	98.69	-	Huang et al. (2016)
Fe-5Ag		7.870	98.73	-	
Fe-10Ag		7.945	98.39	-	
Fe-30Mn	Sintering	5.49	71 ± 3	119 ± 8	Sotoudehbagha et al. (2018)
Fe-30Mn-1Ag		6.2	80 ± 3	156 ± 10	
Fe-30Mn-3Ag		6.92	89 ± 5	174 ± 10	
Pure Fe	Sintering	7.19 ± 0.1	-	63 ± 3	Kupková et al. (2022)
Fe-3.2Cu		6.98 ± 0.1	-	98 ± 3	
Fe-8.0Cu		6.82 ± 0.2	-	161 ± 3	
Pure Fe	Sintering	6.85 ± 0.003	87.04 ± 0.04	101 ± 2	Deng et al. (2021)
Fe-8Cu		6.94 ± 0.002	87.30 ± 0.06	127 ± 1	
Pure Fe	SLM	7.70 ± 0.006	97.84 ± 0.07	≈110	Guo et al. (2021b)
Fe-1.5Cu		7.74 ± 0.003	98.10 ± 0.03	100–150	
Fe-2.3Cu		7.76 ± 0.004	98.35 ± 0.05	100–150	
Fe-7.8Cu		7.86 ± 0.005	98.99 ± 0.06	150–200	
Fe-10.1Cu		7.90 ± 0.002	99.12 ± 0.03	≈400	
Fe-Mn-0.9Cu	As-cast	-	-	138.20 ± 5.26	Mandal et al. (2021)
Fe-Mn-5Cu		-	-	134.20 ± 3.63	
Fe-Mn-10Cu		-	-	158.40 ± 2.88	
Fe-Mn-10Cu-age		-	-	157.20 ± 2.77	
Fe-35Mn-0Cu	Sintering	-	81.58 ± 2	352 ± 5	Mandal et al. (2019)
Fe-34Mn-1Cu		-	81.28 ± 3	330 ± 6	
Fe-32Mn-3Cu		-	80.62 ± 3	315 ± 5	
Fe-30Mn-5Cu		-	79.48 ± 2	330 ± 4	
Fe-25Mn-10Cu		-	77.88 ± 3	233 ± 3	

The improvement of the mechanical properties of pure iron by Ag and Cu elements is mainly attributed to their solid solution strengthening and precipitation strengthening ability. After the addition of Ag and Cu, the iron matrix will form a Cu-rich or Ag-rich second phase due to precipitation (Zhang et al., 2016; Sotoudehbagha et al., 2018; Zhang et al., 2021b; Mandal et al., 2021). These second phases are distributed along the grain boundaries, effectively fill the structural gaps, thereby increasing the overall density and hardness of the alloys (Cao et al., 2006). For instance, adding Ag to the Fe-30Mn alloy enabled the hardness of the alloy to increase from 119 HV of Fe-30Mn to 174 HV of Fe-30Mn-3Ag. At the same time, Fe-30Mn-3Ag also showed three times the shear strength, suggesting that densification and grain refinement can also improve the shear strength of the alloy (Sotoudehbagha et al., 2018). However, it is important to note that the strength of Ag is lower than that of Fe. When the Ag content is too high, the strength of Fe-Ag alloys will decrease (Cao et al., 2006; Huang et al., 2016). Compared with pure iron, only Fe-5Ag alloy in the Fe-x Ag (x = 2, 5, 10 wt%) alloys exhibited better mechanical properties (Huang et al., 2016). As for Cu-containing Fe-based alloys, the changing trend of mechanical properties is similar to Ag-containing Fe-based alloys. Deng et al. stated that the hardness of microwave sintered Fe-8Cu (∼127 HV) was slightly improved compared to the hardness of pure Fe (about 101 HV) (Deng et al., 2021). Guo et al. found out that the Fe-x Cu (x = 0, 1.5, 2.3, 7.8, 10.1 wt%) alloy prepared by SLM exhibited a gradually increasing hardness with the increase in Cu content. What is more, the hardness of the SLMed Fe-10.1Cu alloy increased sharply to 400 HV (Guo et al., 2021b). Similar results were obtained by Mandal et al., suggesting that the addition of 0.9 and 5 wt%Cu did not achieve a significant improvement in the hardness of the alloy. However, when the Cu addition reached 10 wt%, the hardness of the alloy increased significantly (Mandal et al., 2021). The changes in the mechanical properties of the Fe-Mn alloys with the addition of Cu are slightly more complicated. In the study of Mandal et al., as the added amount of Cu increased to 3 wt%, the hardness of Fe-Mn-Cu alloy did not increase but decreased. When the added amount of copper reached 5 wt%, the hardness of the alloy increased. However, when the added amount of Cu reached 10 wt%, the hardness of the alloy decreased again (Mandal et al., 2019). They believed that the decrease in the hardness of the alloy was due to the increase in the accumulation of failure energy (SFE) when a small amount of Cu was added. With the increase in the Cu content, the solid solution strengthening and precipitation strengthening effect of Cu on the alloy overcame the SFE effect and increased the hardness of the alloy (Mandal et al., 2019).

The metal preparation and metal forming processes also have a great influence on the mechanical properties of the alloy. Fe-Cu alloys produced by SLM have high mechanical strength due to their distinctly refined grain structure. However, the microstructures of the iron matrix of all SLMed Fe-xCu (x = 0, 1.5, 2.3, 7.8, 10.1 wt%) alloys are quite compact without any obvious pores (Guo et al., 2021b). In contrast, the Fe-Cu binary alloys produced by sintering have a porous structure closer to the natural bone tissue. With the increase in the Cu content, the size of the alloy pores increases. Although the strength of the alloy is partially lost due to the presence of pores, sintered Fe-Cu alloys can still show acceptably enhanced hardness with the increase in Cu due to the counteracting effect of precipitation hardening (Kupková et al., 2022).

#### 3.2.3 Corrosion Resistance

As mentioned above, the corrosion rate of pure iron is very low and is not suitable for orthopedic implant applications. The alloying treatment and the application of new preparation technology are the main methods to improve the degradation properties of Fe-based materials. Table 8 summarizes the corrosion resistance of Fe-based alloys with antibacterial properties manufactured by different alloying elements and processing processes.

**TABLE 8:** Corrosive properties of Fe-based alloys with antibacterial properties as orthopedic implants.

Alloy composition	Processing method	Medium/solution	Measurement	Ecorr (V)	Icorr (μA/cm^2^)	Corrosion rate (mm/year)	Ref.
Fe-2Ag	Sintering	Hank’s	The electrochemical and immersion tests	−0.84118	10.188	0.1196	Huang et al. (2016)
Fe-5Ag				−0.85577	12.166	0.1403	
Fe-10Ag				−0.89091	15.189	0.1746	
Fe-30Mn	Arc melting	Hank’s	Electrochemical test	−1.11	0.60 ± 0.06	0.007	Liu et al. (2018b)
Fe-30Mn-Ag				−1.10	0.89 ± 0.14	0.012	
Fe-30Mn	Sintering	HBSS	Electrochemical test	−0.213	800	2.61	Sotoudehbagha et al. (2018)
Fe-30Mn-1Ag				−0.303	860	2.49	
Fe-30Mn-3Ag				−0.371	890	2.31	
Pure Fe	Sintering	Hank’s	Potentiodynamic polarization tests	−0.505	48		Kupková et al. (2022)
Fe-3.2Cu				−0.479	57		
Fe-8.0Cu				−0.407	100		
Pure Fe	Sintering	Hank’s	Electrochemical tests	−0.405	29		Deng et al. (2021)
Fe-8Cu				−0.489	59		
Pure Fe	SLM	HBSS	Electrochemical test immersion test	−0.668	19	0.22	Guo et al. (2021b)
Fe-1.5Cu				−0.592	15	0.18	
Fe-2.3Cu				−0.535	26	0.30	
Fe-7.8Cu				−0.515	44	0.51	
Fe-10.1Cu				−0.556	17	0.20	
Fe-Mn-0.9Cu				−0.819 ± 0.065	4.97 ± 0.2	0.058 ± 0.002	Mandal et al. (2021)
Fe-Mn-5Cu	As-cast	HBSS	Static immersion	−0.728 ± 0.0544	4.19 ± 0.3	0.052 ± 0.002	
Fe-Mn-10Cu			Electrochemical corrosion study	−0.789 ± 0.082	4.58 ± 0.1	0.060 ± 0.001	
Fe-Mn-10Cu	As-cast + T6			−0.623 ± 0.066	5.49 ± 0.4	0.072 ± 0.004	
Fe-35Mn-0Cu	Sintering	Hank’s	Potentiodynamic polarization test	−0.678	3.66	1.0922	Mandal et al. (2019)
Fe-34Mn-1Cu				−0.715	2.69	0.8128	
Fe-32Mn-3Cu				−0.718	2.02	0.6096	
Fe-30Mn-5Cu				−0.715	2.88	0.9144	
Fe-25Mn-10Cu				−0.600	20.00	6.5532	

Generally, the addition of antibacterial metal elements, such as Ag and Cu, accelerates the corrosion of Fe-based alloys (Sotoudehbagha et al., 2018; Guo et al., 2021b; Deng et al., 2021; Mandal et al., 2021). The standard electrode potential of Ag (+0.7996 V) and Cu (+0.337V) is much higher than that of Fe (−0.44 V) (Huang et al., 2016). The Cu-containing or Ag-containing second phase with high corrosion potential in Fe-based alloys can be used as independent cathodes, and the iron matrix acts as an anode, forming many micro corrosion cells to accelerate the electrochemical corrosion of Fe-based alloys (Sotoudehbagha et al., 2018). Huang et al. found out that the corrosion rate of Fe-x Ag (x = 2, 5, 10 wt%) alloys increased with increased silver content. Much precipitation of the second phase of sterling Ag brought about by the increase in Ag content significantly accelerated the degradation of the alloys (Huang et al., 2016). For Fe-Cu binary alloys, the corrosion rate varying with copper content is not unidirectional. Guo et al. confirmed that adding a small amount of Cu (1.5 wt%) to pure iron reduced the degradation rate of the alloy compared with pure iron. When the Cu addition reached 2.3 wt%, the alloy showed a significantly increased corrosion rate with the increase in copper addition. The degradation rate of Fe-7.8 wt%Cu alloy (0.51 mm/y) was almost 2.5 times that of pure iron (0.22 mm/y) (Guo et al., 2021b), consistent with the performance trend of sintered Fe-xCu (x = 0, 3.2, 8.0 wt%) alloys prepared by Kupková et al. (2022). However, when the added amount of Cu was further increased to 10.1 wt%, the degradation rate of the alloy reduced to 0.086 mm/y (Guo et al., 2021b) because, in addition to considering the galvanic corrosion induced by the precipitation phase, it is also necessary to pay attention to the influence of the formation of the passivation film and the distribution of the precipitation phase on the degradation of the alloys (Guo et al., 2021b). During the degradation of Fe-Cu alloys, the iron oxide layer forms a passivation film on the surface of the alloys, which will significantly inhibit the continued degradation of the alloys. The deterioration of galvanic corrosion caused by the addition of a small amount of Cu is counteracted by the protective effect of the passivation film. Moreover, the addition of excess Cu makes the alloy surface form more copper-rich phases, and the release of more Cu^2+^ will significantly promote the formation of passivation films. In addition, an excessive Cu-rich phase tends to form a network. The dense reticular copper-rich phase is also a layer of protection of the iron matrix (Guo et al., 2021b). It is not difficult to understand that Fe-based alloys with high Cu content exhibit slow corrosive properties. The corrosion resistance of Fe-Mn-Cu alloys is special. Mandal et al. confirmed that no passivation film is formed during the degradation of Fe-Mn-Cu alloys (Mandal et al., 2021). When the amount of Cu added to the casted Fe-Mn-Cu alloy was 5wt%, the alloy showed improved corrosion resistance due to the formation of a solid solution between Cu and Fe. When the amount of Cu content further increases, the degradation rate will be accelerated due to the intensification of galvanic corrosion (Mandal et al., 2021). The same trend was also found by Mandal et al. The Fe-25Mn-10Cu alloy prepared by powder sintering technology had a corrosion rate of 0.258 mmpy, which was six times that of the Fe-35Mn alloy (Mandal et al., 2019).

The metal preparation and metal forming processes also have a great impact on the corrosive properties of antibacterial Fe alloy. The microwave sintered alloy shows a porous structure. Porous alloys exhibit a larger surface area than as-cast ones with high density (Deng et al., 2021). What is more, Gap erosion is more prone to be developed in porous structures. The degradation rate of microwave sintered Fe-8Cu alloy manufactured by Deng et al. reaches up to 0.69 mm/y (Deng et al., 2021). The space holder method can produce alloys with a highly porous structure. Zhang et al. pointed out that the porosity of FePd2 alloy in this process reaches up to 60%, with a corrosion rate up to 1.162 mm/a (Čapek et al., 2017). Furthermore, the Fe alloy under SLM treatment is confirmed to present with excellent degradable behavior. The SLMed Fe-7.8 Cu alloy exhibits a rapid degradation rate, approximately 2.5 times higher than pure Fe (Guo et al., 2021b). The FePd2 alloy under Spark plasma sintering (SPS) treatment presents a better degradation behavior than the as-cast one because of the grain microstructure. Thus, rational development and utilization of the fabrication process are feasible to the improvement on the degradation behavior of Fe alloys (Čapek et al., 2017).

#### 3.2.4 Biocompatibility

Developing degradable biomaterials with enhanced antibacterial properties is a challenging task because it requires a delicate balance between degradation rate, cell compatibility, and antibacterial properties (Mandal et al., 2021). The addition of Ag, Cu, and other metal elements to iron is conducive to antibacterial efficiency. However, it should be noted that the metal ion concentration released by the alloys should be lower than the cytotoxic limit so that the damage to mammalian cells is minimized (Mandal et al., 2021). Table 9 summarizes the *in vitro* cell compatibility of antibacterial Fe-based alloys with different contents of Ag or Cu.

**TABLE 9:** *In vitro* biocompatibility of Fe-based alloys with antibacterial properties as orthopedic implants.

Alloy composition	Medium	Cell	Culturing time	Test	Result (cell viability)	Ref.
Fe-xAg (x = 2, 5, 10 wt%)	DMEM + 10% FBS	L-929	1, 2, 4 d	CCK-8	Around 100% all the time	Huang et al. (2016)
		VSMCs			Decreased; <70% (4 d)	
		EA. hy-926			Increased (1.2 d); decreased (4 d)	
Fe-30Mn	DMEM + 10% FBS	HUVEC	1, 3, 5 d	MTT	Gradually increased	Sotoudehbagha et al. (2018)
Fe-30Mn-1Ag					Gradually increased	
Fe-30Mn-3Ag					Higher than the other two (1, 3 d)	
Fe-8Cu	DMEM	MG63	1,3 d	CCK-8	slightly decreased, >90% (1, 3 d)	Deng et al. (2021)
Fe-xCu (x = 1.5, 2.3, 7.8, 10.1 wt%)	DMEM + 10% FBS	MG63	1, 5 d	CCK-8	>90%, no significant difference compared to pure iron group (5 d)	Guo et al. (2021b)
Fe-Mn-xCu (x = 0.9, 5, 10 wt%)	MEM + 10% FBS	MG63	4, 12, 24, 72 h	Alamar blue assay	90%–95% (4 h) around 150% (72 h)	Mandal et al. (2021)
	α-MEM + 10% FBS	MC3T3-E1			108%–119% (4, 12 h), significantly increased (24 h), 300% (72 h)	
Fe-(35-x) Mn-xCu (x = 1, 3, 5, 10)	-	MG63	4, 12, 72 h	Alamar blue assay	Fe-Mn and Fe-Mn-Cu: no significant difference	Kupková et al. (2022)
					4–12 h: decreased	
					72 h: increased double	
					All samples (>70%)	

d: days; h: hours.

Antibacterial Fe-based alloys containing Cu or Ag have been reported as non-toxic to L-929 cells (Huang et al., 2016), MG 63 cells (Guo et al., 2021b; Deng et al., 2021; Mandal et al., 2021), and mc3T3-E1 (Mandal et al., 2019) cells. The addition of an appropriate amount of Cu to antibacterial Fe-based alloys has a positive effect on alloy biocompatibility. In Guo et al.’s study, MG63 cells adhered well and developed well on the surface of the SLMed Fe-xCu (x = 1.5, 2.3, 7.8, 10.1 wt%) alloys (Guo et al., 2021b). Fe-xMn-y Cu (x = 35, 34, 32, 30, 25 wt%; y = 0, 1, 3, 5, 10 wt%) alloys also exhibited good biocompatibility, and the extract of alloys showed a significant promotion of MG 63 cell proliferation (Mandal et al., 2019). Cu is an essential microelement that plays an important role in many processes of cellular metabolism (Huang et al., 2016). The positive impact of the addition of Cu on the biocompatibility of Fe-based alloys is not difficult to understand. However, it should be noted that excessive Cu is toxic to cells by promoting the formation of free radicals in cells (Mandal et al., 2021). The Cu content of the reported antibacterial Fe-based alloys is acceptable and does not produce significant cytotoxic effects (Mandal et al., 2019; Guo et al., 2021b; Deng et al., 2021; Mandal et al., 2021; Kupková et al., 2022). *In vitro* biocompatibility of Ag-containing Fe-based alloys is also acceptable. Huang et al. stated that the cell viability of L-929 cells remained at around 100% for 4 days in the extract of sintering Fe-x Ag (x = 2, 5, 10 wt%) (Gao et al., 2019b). Moreover, adding excessive Fe ions was also known to have adverse effects on cell proliferation (Guo et al., 2021b). A recent study confirmed that cell viability could not be inhibited when Fe ion concentrations are below 50 μgmL^−1^ (Kupková et al., 2022; Zhu et al., 2009). Guo et al. showed that, in the extract of SLMed Fe-Cu alloy, the release of Fe ions is within the acceptable range, and the SLMed Fe-Cu alloy had no obvious cytotoxicity to MG63 cells and good cytocompatibility (Guo et al., 2021b).

### 3.3 Zn-Based Alloys With Antibacterial Properties

Zn-based alloys have been increasingly favored as promising orthopedic implants in recent years (Xiao et al., 2021a; Zhang et al., 2021b). As an essential trace element, Zn is involved in the formation of bone and has perfect biocompatibility (Solomons, 2013). The antibacterial activity of Zn has been corroborated (Zhao et al., 2016a; Bakhsheshi-Rad et al., 2017). What is more, Zn is more dominant compared to Mg and Fe because the degradation rate of Zn is between that of Mg and Fe, and degradation products can be fully absorbed (Bowen et al., 2013). Nevertheless, the poor mechanical properties of pure Zn fail to meet the requirements for orthopedic implants (Hernández-Escobar et al., 2019; Peng et al., 2021b). Besides, cytotoxicity is prone to be induced due to a high concentration of Zn ions by inhibiting ECM mineralization (Li et al., 2019a; Wang et al., 2021b). Thus, a growing number of studies have focused on alloying Zn-based materials to ameliorate mechanical properties and biocompatibility (Li et al., 2019b; Hernández-Escobar et al., 2019). Recently, with the antibacterial effect of implants receiving much more attention, several Zn-based alloy orthopedic implants with antibacterial properties have been reported.

#### 3.3.1 Antibacterial Properties

Zn-Ag alloys are the most studied Zn alloys with antibacterial activity. As expected, Zn-Ag alloys have been verified to be promising *in vitro* antibacterial activity against Gram-negative bacteria (*E. coli*) (Xie et al., 2018), Gram-positive and multi-resistant bacteria, including a potential strain of infection after maxillofacial surgery with an intraoral approach called *Streptococcus gordonii* (*S. gordonii*) (Loo et al., 2000; Li et al., 2018a), *S. epidermidis*, *S. aureus*, MRSA, and methicillin-resistant *Staphylococcus epidermidis* (MRSE) (Qu et al., 2021). Similar to Mg-Ag alloys, the antibacterial properties of Zn-Ag alloys enhance gradually with an increase in the Ag content (Xie et al., 2018; Qu et al., 2021). It is worth noting that the Zn-2Ag alloy demonstrated significant *in vivo* antibacterial activity against MRSA and inhibition of osteomyelitis in the rat femoral osteomyelitis prevention model (Qu et al., 2021). In addition, porous Zn-Ag alloy exhibited a stronger antibacterial effect than bulk Zn-Ag alloy (Xie et al., 2018). Given the biomimetic effect and osteogenic ability of porous structures, this finding adds further evidence and motivation for the development of porous alloy implants.

Other studies attempt to further add other alloying elements to manufacture ternary and quaternary Zn-Ag alloys, to improve the performance of alloy implants. Given that Mg is the most effective element to enhance the comprehensive performance of Zn-based materials among numerous alloying elements (Venezuela and Dargusch, 2019), Xiao et al. added Mg to the Zn-Ag alloy and developed Zn-0.05Mg-1.0Ag alloy with both superior mechanical properties and antibacterial capacity. This kind of ternary alloy exhibits a powerful antibacterial ability against *S. aureus* and *E. coli* (over 99%) (Xiao et al., 2019). Similarly, the Zn-0.04Mg-2Ag alloy prepared by Wu et al. also has strong antibacterial properties against *S. aureus* and *S. epidermidis*. However, in their study, the inhibitory effect of the alloy on *E. coli* is weak (Wu et al., 2021). The reason for such a discrepancy requires further studies and explanation. In addition, the quaternary Zn-2Ag-1.8Au-0.2V(wt%) alloy demonstrated enhanced antibacterial behaviors against *S. gordonii*, which was manufactured with the antibacterial ability of Ag (Li et al., 2019c). Combined with the antibacterial ability of Ag and Zr, the Zn-1Ag-0.05Zr alloy revealed ascendant inhibitory action against *E. coli* and *S. aureus* (Wątroba et al., 2019). However, it should be noted that the antibacterial ability of this ternary alloy seemed to be weaker than that of the Zn-1Ag alloy. This might be attributed to the low degradation rate and its impact on ions releasing, which is crucial for the generation of antibacterial ability (Wątroba et al., 2019). More studies are clearly required to fully understand this phenomenon.

Zn-Cu alloys are also confirmed to have great potential as orthopedic implants with antibacterial properties. They have been confirmed effectively against Gram-negative (*E. coli*) (Shuai et al., 2020), Gram-positive, and drug-resistant strains (*S. aureus*, S*. epidermidis*, MRSA, and MRSE) (Qu et al., 2020). The antibacterial ability of the alloy is proportional to the Cu content (Qu et al., 2020; Shuai et al., 2020). In addition, Li et al. demonstrated the significant inhibitory effect of Zn-4Cu alloy on the biofilm formation of mixed oral bacteria, the main causative agents of craniomaxillofacial osteosynthesis (Li et al., 2019d). Zn-Cu also exhibited excellent *in vivo* antibacterial properties. In a rat femoral intramedullary nail MRSA infection model, the Zn-2Cu alloy implants suppressed inflammation and toxicities caused by MRSA and played a beneficial role in preventing infection-related bone loss (Qu et al., 2020). Further alloying and processing on Zn-Cu alloys led to satisfactory outcomes. For instance, the ternary Zn-1Cu-0.1Ti alloy presented significantly improved antibacterial properties, which are manufactured by adding Ti element and dealing with hot rolling and cold rolling (Lin et al., 2020). The ternary Zn-0.5Cu-0.2Fe alloy guided bone regeneration (GBR) films obtained by introducing the Fe element and dealing with hot extrusion showed extensive inhibition of *S. gordonii* and mixed oral bacteria (Zhang et al., 2021c). Moreover, the Zn-Cu bimetallic foam obtained by electrochemical deposition and subordinate diffusion heat treatment also has excellent antibacterial performance (Tong et al., 2020). Besides, the unique porous architecture of this bimetallic foam plays a critical role in osseointegration and vessel ingrowth. By adjusting the pore structure, orthopedic implants with different properties and suitable for different body sites can also be customized (Tong et al., 2020). Overall, the application prospect of Zn-Cu alloys and Cu-containing zinc alloys prepared by a special process in orthopedic implants is very broad.

In addition, it has been reported that Zn-0.5Mg alloy shows a good antibacterial effect on *E. coli* and *S. aureus* (Xiao et al., 2018). The Zn-0.8Mg-0.2Sr alloy prepared by Capek et al. exhibited inhibitory effects on the adhesion and biofilm formation of *S. gordonii* (Čapek et al., 2021). Moreover, Bakhsheshi-Rad et al. confirmed that the addition of Mg element to Zn-Al alloys could improve the antibacterial properties of the alloys, which was expected to be further improved with higher Mg content (Bakhsheshi-Rad et al., 2017). As can be seen, Mg is an alloying element that is worthy of attention for fabricating Zn alloys with antibacterial properties.

From the current status of research, the development of Zn-based alloy orthopedic implants with antibacterial is still in the elementary stage, and most studies are primarily concentrated on the application of classical antibacterial metal elements, such as Ag and Cu (Table 6). However, there is no doubt that Zn-based alloys hold great potential as orthopedic implants with antibacterial properties. The research on processing technology and alloying elements, especially those with antibacterial properties, will promote the usage of Zn alloys in the field of orthopedic implants.

#### 3.3.2 Mechanical Properties

As load-bearing implants, pure Zn exhibits poor behavior on mechanical strength and stretchability (Xiao et al., 2020). It has been shown that the tensile strength of pure Zn ranges from 10–110 MPa, elongation is 0.32%–36%, and Vickers hardness is 38–39 HV1 (Li et al., 2018b). The mechanical properties of alloys can be significantly improved by adding alloying elements and the fabrication process (Xiao et al., 2020). Parameters on mechanical properties of antibacterial Zn alloys are summarized in Table 10.

**TABLE 10:** Mechanical properties of Zn-based alloys with antibacterial properties as orthopedic implants.

Alloy composition	Processing method	Yield strength (MPa)	Ultimate tensile strength (MPa)	Elongation at fracture (%)	Hardness (HV)	Ref.
**Zn-Ag alloys**
Zn-2.5Ag	As-cast + 410 °C/6 and 12 h + extrusion	157	203	32–36	-	Sikora-Jasinska et al. (2017)
Zn-5.0Ag		-	-		-	
Zn-7.0Ag		236	287		-	
Zn-4Ag	Thermomechanical treatment	157	261	37	73	Li et al. (2018b)
	Additional precipitation hardening	149	215	24	82	
Zn-1Ag-0.05Zr	As-cast + 400 °C/4 h + hot extrusion	168 ± 3	211 ± 1	35 ± 1	-	Wątroba et al. (2019)
Zn-Ag-Au-V	Thermomechanical treatment	129	231	59	61	Li et al. (2019c)
	Thermomechanical treatment + additional precipitation hardening	168	233	17	96	
**Zn**-**Cu alloys**						
Zn-2Cu	Extrusion	226	270	41	-	Qu et al. (2020)
Zn-3Cu	As-cast	95 ± 2	98 ± 3	1.2 ± 0.3	79.8 ± 2.0	Lin et al. (2021)
	Hot-rolled	189 ± 4	244 ± 4	38.4 ± 0.8	79.0 ± 1.3	
	Hot-rolled + cold-rolled	193 ± 3	268 ± 3	66.4 ± 0.9	62.0 ± 1.4	
Zn-4Cu	As-cast	73.0	105.4	3.4	-	Li et al. (2019e)
	As-rolled	327.1	393.3	38.8	-	
Zn-4Cu	Laser powder bed fusion	165 ± 7	207 ± 7	22.5	97 ± 2	Shuai et al. (2020)
						
Zn-Cu foam	Electrochemical deposition + diffusion heat treatment	12.1 ± 1.8	-	-		Tong et al. (2020)
Zn-1Cu-0.1Ti	As-cast	86.1 ± 2.6	92.4 ± 4.4	1.4 ± 0.8		Lin et al. (2020)
	Hot-rolled	175.4 ± 3.8	205.7 ± 5.5	39.2 ± 1.4		
	Hot-rolled + cold-rolled	204.2 ± 4.3	249.9 ± 3.8	75.2 ± 1.9		
Zn-3Cu-0.2Ti	As-cast	117 ± 3	124 ± 3	1.2 ± 0.3	83.8 ± 1.8	
	Hot-rolled	224 ± 3	290 ± 4	42.7 ± 1.1	80.6 ± 1.5	
	Hot-rolled + cold-rolled	211 ± 3	271 ± 5	72.1 ± 1.6	63.6 ± 1.0	
Zn-0.5Cu-0.1Fe	Hot extrusion	115.7	176.0	43.9	-	Zhang et al. (2021c)
Zn-0.5Cu-0.2Fe		152.3	202.3	41.2	-	
Zn-0.5Cu-0.4Fe		182.1	240.1	20.5	-	
**Zn**-**Mg alloys**						
Zn-0.05 Mg	As-cast + extrusion	160	225	26	-	Xiao et al. (2018)
Zn-0.04Mg-2Ag	Template replication technique	7.82	-	-	-	Wu et al. (2021)
Zn-0.05Mg-0.5Ag	As-cast + homogenization + extrusion	224	-	-	58	Xiao et al. (2020)
Zn-0.05Mg-1Ag		234	-	-	67	
Zn alloy (Zn 95%, Mg 0.001%–2.5%,Fe 0.01%–2.5%)	As-cast	150–340	160–380	15–49	80–110	Wang et al. (2019)
Zn-0.8Mg-0.2Sr	As-cast + homogenization annealing+ extrusion	244 ± 1	324 ± 1	20 ± 1	98 ± 1	Capek et al. (2021)
**Zn**-**Mn alloys**						
Zn-0.8Mn	As-cast + hot extrusion	126.7 ± 2.4	218.6 ± 0.5	64.2 ± 4.4	-	Shi et al. (2019)
Zn-0.8Mn-0.4Ag		156.1 ± 6.0	251.3 ± 7.3	62.6 ± 4.2	-	
Zn-0.8Mn-0.4Cu		191.3 ± 4.1	308.3 ± 0.6	38.9 ± 5.4	-	
Zn-0.8Mn-0.4Ca		253.4 ± 1.3	343.2 ± 1.6	8.0 ± 1.4	-	
						
**Zn**-**Al alloys**						
Zn-0.5Al	As-cast	-	79 ± 2	1.5 ± 0.1	71 ± 2	Bakhsheshi-Rad et al. (2017)
Zn-0.5Al-0.1 Mg		-	-	-	79 ± 3	
Zn-0.5Al-0.3 Mg		-	93 ± 3	1.7 ± 0.1	87 ± 3	
Zn-0.5Al-0.5 Mg		-	102 ± 4	2 ± 0.1	94 ± 4	
**Zn**-**Ce alloys**						
Zn-2Ce	Laser additive manufacturing technique	180.6 ± 7.1	247.4 ± 7.2	7.5%	-	Yang et al. (2021b)

It is reported that the addition of Ag and Cu can not only activate slip systems to maintain preponderant elongation of Zn alloys (Sikora-Jasinska et al., 2017; Lin et al., 2020) but also contribute to solution strengthening and effective grain refinement for the enhancement of mechanical behavior (Wątroba et al., 2019; Qu et al., 2020). Based on the Hall–Petch strengthening mechanism, the smaller particle size alloys are, the stronger yield strength they present (Lin et al., 2020). Precipitation of AgZn3 and ε-CuZn5 is a critical factor in improving the hardness and strength of alloys (Xie et al., 2018; Li et al., 2019e; Wątroba et al., 2019; Lin et al., 2020). When the Ag content reaches 3.5%, porous Zn-3.5Ag scaffold precipitates secondary phase AgZn3 (Xie et al., 2018), which decreases the grain size and helps further grain refinement (Wątroba et al., 2019). Nevertheless, the improvement in mechanical properties of alloys is not apparently observed when the size of precipitate phases reaches a certain level. Shuai et al. fabricated the Zn-Cu alloy by laser powder bed fusion, finding that when the Cu content was up to 4 wt%, the mechanical behavior of alloys appeared to be slightly decreased due to the stress concentration of the ε-CuZn5 phase in a larger size (Shuai et al., 2020). Moreover, Shi et al. suggested that although adding Cu increased the mechanical properties substantially, its stretchability declined from 64.2% to 38.9% compared with the Zn-0.8Mn alloy. However, the Cu alloy shows sufficiently high ductile properties (Shi et al., 2019). As a commonly used alloying element, Mg is also added to the antibacterial Zn alloy to enhance mechanical properties (Xiao et al., 2018). Similar to the effects of Cu and Ag, adding Mg remarkably improves the mechanical properties of antimicrobial Zn alloy mainly because of the formation of solid solution, grain refinement, and the obstacle of grain boundary sliding by intermetallic particles (Bakhsheshi-Rad et al., 2017; Čapek et al., 2021). During this process, the fine Mg_2_Zn_11_ particles with uniform distribution are pivotal in the excellent hardness and plasticity of the antibacterial Zn-based alloys (Xiao et al., 2018). Furthermore, the addition of alloying elements such as Ti (Lin et al., 2021), Ca (Shi et al., 2019), and Ce (Yang et al., 2021b) is proved to be effective in improving the antibacterial Zn alloy.

Besides adding alloying elements, improving mechanical properties can also be realized by proper fabrication processes such as extrusion, rolling, forcing, and annealing (Li et al., 2018b). The same as the addition of alloying elements, the purpose of these processes is all grain refinement, thus obtaining alloys with higher strength (Wątroba et al., 2019). As one of the most commonly used fabrication processes, extrusion has been extensively exploited for its drastic improvement of the mechanical properties of alloys (Sikora-Jasinska et al., 2017; Xiao et al., 2018; Čapek et al., 2021). Sikora-Jasinska et al. fabricated several kinds of binary Zn-x Ag alloys (x = 2.5, 5.0, 7.0 wt%) by hot extrusion (Sikora-Jasinska et al., 2017). Microscopic analysis indicated that hot extrusion brought a marked decrease in the grain size of alloys. The higher the Ag content, the smaller grain. What is more, yield strength and ultimate tensile strength of alloy were improved (Zn-7.0%Ag alloy corresponded to 236 and 287 MPa, respectively) due to the precipitation of fine AgZn3 particles along grain boundaries (Sikora-Jasinska et al., 2017). Similar results are seen in the other Zn alloys by hot extrusion (Li et al., 2018b; Xiao et al., 2018; Kodetová et al., 2019; Čapek et al., 2021). This may be related to the occurrence of dynamic recrystallization (DRX) during the extrusion process. Capek et al. found out that the microstructure of extruded materials consisted of complete recrystallized grains with a size of merely 2.4 μm (Čapek et al., 2021). Besides significant enhancement of the strength of alloys, hot extrusion can also eliminate the fragility of as-cast alloys and improve plasticity. Shi et al. suggested that the elongation of extruded Zn-0.8Mn alloys arrived at 64.2%, while it was solely 1.0% for as-cast ones (Xiao et al., 2018). The solution heat-treatment is also an approach for enhancing the mechanical properties of alloys (Sun et al., 2020). By this process, Sun et al. manufactured the Zn-0.8Mn alloy and found that solution heat-treatment at 380°C enabled MnZn_13_ particles to dissolve into the Zn matrix, leading to a solid solution hardening effect. This effect appeared to become more significant with elongated treatment time (Sun et al., 2020). This is likely because heat treatment (solution annealing) transforms a dendritic cast structure into a globular structure, resulting in a more stable structure, unlike the dendritic presence of anisotropy in as-cast alloys (Kodetová et al., 2019). Rolling is also a frequent process. Lin et al. compared Zn-Cu alloy under the treatment of hot and cold rolling with the as-cast and hot-rolled one, finding a significant enhancement in strength and plasticity because, with the process of hot and cold rolling, precipitated hard and brittle ε- CuZn_5_ particles were evenly distributed in the η-Zn matrix after fragmentation into minute particles (Lin et al., 2020). Nonetheless, processing in such a manner leads to the reduction in the Cu content in the η-Zn matrix and thus a weakening of solid-solution strengthening, causing a dramatic decline in hardness (Lin et al., 2020; Lin et al., 2021). Furthermore, 3D printing and additive manufacturing are also suited to the fabrication of Zn alloys with outstanding mechanical properties (Shuai et al., 2020; Yang et al., 2021b).

Alloys with porous structures offer desirable structural conditions for the proliferation and differentiation of osteoblasts because their pore-size range is consistent with that of the cancellous bone pore (400–600 mm) in the human body (Wu et al., 2021). What is more, the unique degradable characteristics enable bone healing without subsequent operating surgeons after implantation (Tong et al., 2020). For this reason, the development of porous structure quickly gains popularity for antibacterial Zn and its alloys as bone implants. Compared with bulk structure, the mechanical behavior of porous structure (compressive plateau stress and elastic modulus) gets poor because of increased porosity, which restricts its high load-bearing applications (Zhao et al., 2016b). The current fabrication of porous scaffolds mainly concentrates on pure Zn (Zhao et al., 2016b; Zhao et al., 2018; Cockerill et al., 2020). In contrast, only Zn-0.04Mg-2Ag is successfully developed and proved with excellent biological performance among porous antimicrobial Zn alloys as bone implants (Wu et al., 2021). Thus, the fabrication of more porous scaffolds of antibacterial Zn alloys represents new research directions in the future.

#### 3.3.3 Corrosion Resistance

Compared with Mg and Fe, Zn has moderate corrosion resistance, because its standard corrosion potential (−0.762 VSCE) ranges between Fe (−0.440 VSCE) and Mg (−2.372 VSCE) (Li et al., 2018b), which avoids hydrogen accumulation caused by rapid corrosion rate and strong corrosion resistance to hinder clinical applications of alloys. Nonetheless, the corrosion rate of pure Zn is 9.6 μm/a (Wątroba et al., 2019), and there is a clinical need for adding alloying elements to improve the degradation rate. Table 11 summarizes the corrosion resistance performance of antibacterial Zn alloys in *in vitro* studies.

**TABLE 11:** Corrosion resistance of Zn-based alloys with antibacterial properties as orthopedic implants.

Alloy composition	Processing method	Solution	Measurement	Ecorr (V)	Icorr (μA/cm^2^)	Corrosion rate (μm/year)	Ref.
**Zn-Ag alloys**
Zn-2.5Ag	As-cast + 410 °C/6 and 12 h + extrusion	Hanks’ modified solution	Potentiodynamic polarization test	−1.12 ± 0.01	9.2 ± 0.9	137 ± 21	Sikora-Jasinska et al. (2017)
Zn-5.0Ag				−1.12 ± 0.02	9.7 ± 0.7	144 ± 7	
Zn-7.0Ag				−1.14 ± 0.04	9.9 ± 0.6	147 ± 18	
Zn-1Ag- 0.05Zr	As-cast + 400 °C/4 h + hot extrusion	Hank’s	Electrochemical test	−1.008 ± 0.004 (vs. Ag/AgCl)	4.6 ± 2.2	76.9 ± 33.3	Wątroba et al. (2019)
Zn-Ag-Au-V	Hot rolling	DPBS	Immersion test	-	-	7.34 ± 0.64	Li et al. (2019c)
**Zn**-**Cu alloys**							
Zn-3Cu	As-cast	Hank’s	Electrochemical test	−0.932 ± 0.157	14.3 ± 0.6	190 ± 8	Lin et al. (2021)
	Hot-rolled			−0.946 ± 0.119	19.2 ± 0.4	255 ± 5	
	Hot-rolled + cold-rolled			−0.979 ± 0.185	23.4 ± 0.7	311 ± 9	
Zn-4Cu	As-cast	HBSS	Electrochemical test	-	-	140–230	Li et al. (2019e)
	As-rolled			-	-	130–190	
Zn-4Cu	Laser powder bed fusion	SBF	Electrochemical test	-	12.88 ± 0.59	190	Shuai et al. (2020)
Zn-Cu foam	Electrochemical deposition+ diffusion heat treatment	Hank’s	Electrochemical polarization testing	−0.951 ± 0.105 (vs. SCE)	13.4 ± 0.5	177.3 ± 7.3	Tong et al. (2020)
Zn-1Cu-0.1Ti	As-cast	Hank’s	Immersion test	−1.025 ± 0.264 (vs. SCE)	21.5 ± 0.4	315 ± 6	Lin et al. (2020)
	Hot-rolled			−1.123 ± 0.185	111.2 ± 0.9	1,628 ± 13	
	Hot-rolled + cold-rolled			−1.100 ± 0.201	67.7 ± 0.5	991 ± 7	
Zn-3Cu-0.2Ti	As-cast			−0.961 ± 0.204	10.9 ± 0.4	145 ± 5	
	Hot-rolled			−0.982 ± 0.189	19.0 ± 0.5	252 ± 7	
	Hot-rolled + cold-rolled			−0.993 ± 0.172	22.5 ± 0.8	299 ± 11	
Zn-0.5Cu- 0.1Fe	Hot extrusion	α-MEM/artificial saliva	Electrochemical test	−1.12/−0.87	17.82/2.38	-	Zhang et al. (2021c)
Zn-0.5Cu- 0.2Fe				−0.92/−0.89	16.78/3.02	42.4	
Zn-0.5Cu- 0.4Fe				−0.93/−0.91	16.43/5.29	50.4	
**Zn**-**Mg alloys**							
Zn-0.05 Mg	Hot extrusion	SBF	Immersion test	-	-	150	Xiao et al. (2018)
Zn-0.8Mg- 0.2Sr	As-cast + homogenization annealing + extrusion	PS/SBF	Electrochemical test	-	-	55/8.5	Capek et al. (2021)
**Zn**-**Mn alloys**							
Zn-0.8Mn	As-cast	Hank’s	Immersion test	−1.08 ± 0.01	9.53 ± 1.79	145 ± 27	Sun et al. (2020)
	As-cast + T4 (380 °C/15 h)			−1.07 ± 0.01	8.09 ± 0.87	123 ± 11	
	As-cast + T4 (380 °C/45 h)			−1.08 ± 0.01	6.25 ± 1.20	95 ± 18	
Zn-0.8Mn	Hot extrusion	SBF	Electrochemical test	−1.07 ± 0.02	6.76 ± 0.35	101 ± 9	Shi et al. (2019)
Zn-0.8Mn- 0.4Ag				−1.19 ± 0.01	11.22 ± 0.79	168 ± 21	
Zn-0.8Mn- 0.4Cu				−1.18 ± 0.01	8.91 ± 0.67	133 ± 0.015	
Zn-0.8Mn- 0.4Ca				−1.16 ± 0.01	10.72 ± 1.28	160 ± 19	
**Zn**-**Al alloys**							
Zn-0.5Al	Cast	Kokubo solution	Electrochemical test	-	-	150 ± 10	Bakhsheshi-Rad et al. (2017)
Zn-0.5Al- 0.1 Mg				-	-	130 ± 10	
Zn-0.5Al- 0.3 Mg				-	-	-	
Zn-0.5Al- 0.5 Mg				-	-	110 ± 10	
**Zn**-**Ce alloys**							
Zn-1,2,3Ce	Laser additive manufacturing technique	SBF	Immersion test	−1.02–1.11	6.97	24.2 ± 1.1	Yang et al. (2021b)

SBF: simulated body fluid; HBSS: Hank’s balanced salt solution; DPBS: Dulbecco’s Phosphate-Buffered Saline; PS: physiological saline solution.

The addition of the Ag and Cu elements remarkably decreases the corrosion resistance of Zn alloys, thus substantially improving the degradation rate (Li et al., 2018b; Qu et al., 2021) because possibly a higher standard electrode potential of Ag and Cu than Zn declines the Ecorr value (Shi et al., 2019), and the formation of AgZn_3_ and −CuZn_5_ phases induces galvanic corrosion (Qu et al., 2020; Shuai et al., 2020), which is confirmed to be more apparent in the samples with high Cu contents (Zn-7.0Ag, Zn-4.0Cu) (Sikora-Jasinska et al., 2017; Shuai et al., 2020). The AgZn_3_ phase can damage the densified surface of the matrix (Xie et al., 2018), such as ZnO, Zn(OH)_2_, and Ca_3_(PO_4_)_2_ (Sikora-Jasinska et al., 2017; Wątroba et al., 2019), thereby further accelerating the degradation rate. However, it is not absolute. The Zn-Ag-Au-v alloy exhibits a lower degradation rate than pure Zn in the test carried out in phosphate-buffered saline (PBS) due to the formation of zinc phosphate, a more densified passivation film than Zn(OH)_2_ (Kodetová et al., 2019). Unlike the AgZn_3_ phase, the ε- CuZn_5_ phase is demonstrated to have twofold implications on the corrosion behavior. The continuous reticular structure formed by the ε- CuZn_5_ phase in the matrix functions as a protective barrier to hinder corrosion (Lin et al., 2020). Adding other elements exerts a certain influence on the degradation rate of alloys. For instance, the addition of 0.2 wt% Fe to Zn-0.5Cu alloys accelerates the degradation of alloys (Zhang et al., 2021c). Ti (Lin et al., 2021), Ce (Yang et al., 2021b), and Mn (Sun et al., 2020) are also confirmed to enhance corrosion resistance when added to antibacterial Zn alloys. In contrast, the corrosion resistance of Zn alloys is not particularly affected by adding trace amounts of Mg. The corrosion rate is approximately 0.15 mm/a, consistent with that of pure Zn (Xiao et al., 2018).

The fabrication process also exerts a dramatic effect on the corrosion rate. Compared to as-cast alloys, hot rolled ones present more uniform corrosion characteristics due to grain refinement and even distribution (Li et al., 2019e; Kodetová et al., 2019). Furthermore, hot rolling tends to accelerate corrosion, which may be related to the galvanic corrosion occurring between secondary phases and the Zn matrix, as well as the destruction of the natural oxide layer (Li et al., 2019e) and reticular structure formed by secondary phases in the matrix (Lin et al., 2021). In the study on Zn-3Cu and Zn-3Cu-0.2Ti alloys by Lin et al., compared with hot rolling, the hot-rolled + cold-rolled samples exhibited a more rapid corrosion rate due to the higher content of ε-CuZn_5_ secondary phases and increased micro-battery reaction with η-Zn phases in the matrix (Lin et al., 2021). However, the results of the alloy foam with porous structure appear to be in contrast to those of bulk alloys. The Zn-Cu foam alloy manufactured by Tong et al. is proven with excellent corrosion properties (Tong et al., 2020). Nyquist plots show that the radius of the capacitive arc of foam increases after heat treatment, leading to larger corrosion resistance and a lower corrosion rate (Tong et al., 2020).

Ideally, the strength of antimicrobial Zn alloy *in vivo* as implants diminishes over time, which is crucial for reducing stress shielding and recovering the physiological stress of bones (Wang et al., 2019). Thus, *in vivo* studies on biodegradable materials are critical. Nevertheless, recent *in vivo* studies on the biodegradation of Zn alloys are rare (Wang et al., 2019). Clearly, future studies will need to focus on degradation and changes in mechanical properties of alloys *in vivo* to obtain antibacterial Zn alloys with outstanding comprehensive performance as bone implants.

#### 3.3.4 Biocompatibility

As bone implants, the evaluation of the biocompatibility of antibacterial Zn-based alloys has major clinical implications (Wu et al., 2021). According to the reference to the human body, the recommended intake levels of Zn, Cu, Mg, and Ag are 12–16 Mg/d, 0.9–1.2 Mg/d, 240–400 Mg/d, and 0.4–27 μg/d (Xiao et al., 2020; Lin et al., 2021). There are no toxic side effects to human tissues and organs when the amount of ions release is lower than that of the daily recommended intake. Otherwise, over-releasing of metal ions tends to trigger cytotoxicity, and then inflammation response, carcinogenic stimulation, autoimmunity, and allergy (Xiao et al., 2020). Currently, studies on the biocompatibility of Zn alloys mainly focus on *in vitro* analysis. Table 12 summarizes the biocompatible performance of antibacterial Zn alloys in *in vitro* studies.

**TABLE 12:** *In vitro* biocompatibility of Zn-based alloys with antibacterial properties as orthopedic implants.

Alloy composition	Processing method	Measurement	Cell line	Result	Ref.
**Zn-Ag alloys**
Zn-4.0Ag	Thermomechanical treatment	XTT assay	L929 Saos-2	The alloy showed a certain degree of toxicity	Li et al. (2018b)
		BrdU assay			
Zn-Ag-Au-V	Thermomechanical treatment	XTT assay	L929 Saos-2	The alloy showed acceptable toxicity with cells exposed to 10% and 16.7% extracts	Li et al. (2019c)
		BrdU assay			
**Zn**-**Cu alloys**					
Zn-2Cu	Extrusion	CCK-8, live/dead cell staining, cytoskeletal staining	MC3T3-K	The cytocompatibility was improved compared to pure Zn. The cells in the Zn-2Cu alloy showed a substantial degree of spreading, and the red tensile filaments composed of actin were fully spread	Qu et al. (2020)
Zn-4Cu	Hot rolling	CCK-8, BrdU assay	L929, Saos-2, TAg	There was no apparent cytotoxic effect	Li et al. (2019e)
Zn-4Cu	Laser powder bed fusion	CCK-8	MG-63	It presented favorable biocompatibility	Shuai et al. (2020)
Zn-Cu foam	Electrochemical deposition + diffusion heat treatment	CCK-8	MC3T3-E1	A 12.5% concentration of the extract showed >90% cell viability	Tong et al. (2020)
Zn-1Cu-0.1Ti	As-cast	CCK-8	MC3T3-E1	The cell viability of both exceeded 90% after culturing for 1 d, indicating good, *in vitro* cytocompatibility	Lin et al. (2020)
			MG-63		
Zn-0.5Cu- xFe (x = 0.1, 0.2, 0.4 wt%)	Hot extrusion	CCK-8, BrdU assay	L929, Saos-2, TAg	There were no apparent cytotoxic effects	Zhang et al. (2021c)
**Zn**-**Mg alloys**					
Zn-0.05 Mg	Hot extrusion	Relative growth rate (RGR)	L929	There was little toxicity to the general functions of the animal	Xiao et al. (2018)
Zn-0.04 Mg- 2Ag	Template replication technique	CCK-8, cytoskeleton staining	MC3T3	The scaffold had excellent biocompatibility and fine osteogenic induction	Wu et al. (2021)
Zn-0.05 Mg- xAg (x = 0, 0.5, 1 wt%)	As-cast + homogenization + extrusion	Relative growth rate (RGR)	L929	The hemolysis rates were within the safe range, and the alloys were safe	Xiao et al. (2020)
Zn-0.8 Mg- 0.2Sr	As-cast + homogenization annealing + extrusion	Live/dead fluorescence staining, CCK-8, BrdU assay	L929, Saos-2, TAg	The extracts diluted to 25% had no adverse effects toward cells	Capek et al. (2021)
**Zn**-**Mn alloys**					
Zn-0.8 Mn	Hot treatment	CCK-8	L929, rBMSCs	Poor L929 cell viability of 8% by As-cast jumps to be about 100% by hot-treatment for cultivating 24 h. Good rBMSCs viability was insensitive to the hot-treatment	Sun et al. (2020)
Zn-0.8 Mn- 0.4x (x = Ag, Cu, or Ca)	Hot extrusion	MTT	L929	Addition of Cu or Ca much alleviated cytotoxic potential of Zn-0.8Mn alloy	Shi et al. (2019)
Zn-0.5 Al- xMg (x = 0.1, 0.3, 0.5 wt%)	As-cast	MTT	MC3T3	The results of cytotoxicity demonstrated that the Zn-0.5Al-0.5 Mg alloy was biocompatible	Bakhsheshi-Rad et al. (2017)
Zn-xCe (x = 1%, 2%, 3%)	Laser additive manufacturing technique	CCK-8	MG-63	There was no obvious toxicity to MG-63 cells	Yang et al. (2021b)

XTT, assay: tetrazolium assay; BrdU assay: bromodeoxyuridine assay.

The addition of Ag (Li et al., 2018b) and Cu (Shi et al., 2019; Shuai et al., 2020) substantially increases the biocompatibility of pure Zn without producing extra cytotoxicity and even exhibits a significant improvement in the cell metabolic and proliferative activity (Li et al., 2018b; Li et al., 2019e). Xiao et al. analyzed the cell morphology of L-929 and relative growth rate (RGR), confirming that adding 0.5 and 1 wt% Ag to the Zn-0.05wt.%Mg alloy largely increased RGR (Xiao et al., 2020). Another study demonstrated that the cellular survival rate exceeded 80% when the concentration in Zn-3Cu and Zn-3Cu-0.2 Ti alloy extracts was no more than 25% (Lin et al., 2021). Except for the concentration of Zn and Cu ions in extracts within the recommended amounts, it is also correlative with the high cellular tolerance of ions (Li et al., 2019e; Zhang et al., 2021c). The Zn-Mg alloy is a non-toxic material with good biocompatibility. Its extracts show excellent cell morphology, tolerance, and adherence (Wang et al., 2019; Wu et al., 2021; Čapek et al., 2021). What is more, the *in vivo* degradation of alloys causes no harm to important organs and cellular structures (Xiao et al., 2018). However, it should be noted that extracts without dilution usually exhibit apparent cytotoxicity, which adversely affects the survival, proliferation, and adherence of cells. This is mainly due to the inhibition of high ions concentration and high osmolarity on cellular adherence and growth (Lin et al., 2021). Generally, metal ions in low concentrations are beneficial to cells, but an opposite trend is observed in high concentrations (Shi et al., 2019; Sun et al., 2020). In undiluted extracts of Zn-4Ag (Li et al., 2018b) and Zn-Ag-Au-V alloys (Kodetová et al., 2019), apparent cytotoxicity is observed, which almost completely inhibits the cellular activity and proliferation. The dilution of extracts shows no toxicity to cells, enabling a gradual increase in cellular activity (Kodetová et al., 2019; Lin et al., 2020; Tong et al., 2020).

Zn is confirmed with osteogenesis activities, promoting bone formation by increasing calcium content, collagen content, and alkaline phosphate activity (Xiao et al., 2018). The addition of Cu, Ag, and Mg elements enables the further proliferation of osteoblasts, thus presenting better osteogenic induction in antibacterial Zn alloys (Xiao et al., 2018; Qu et al., 2020; Wu et al., 2021). Mg ions significantly upregulate the expression of OSX, OPN, and OC9 (Wu et al., 2021), leading to more adhesion and proliferation of cells, thereby promoting bone healing (Xiao et al., 2018). Similarly, releasing Cu ions enhances the expression of osteogenesis-related genes ALP, COL 1, OCN, and Runx-2 (Qu et al., 2020).

Currently, there is not much *in vivo* research on the biosafety of antimicrobial Zn alloys, most of which are focused on Zn-Mg alloys (Xiao et al., 2018; Wang et al., 2019; Xiao et al., 2021b). Nonetheless, due to the differences in the degradable environment and sensitivity (Xiao et al., 2018), there is an apparent discrepancy between *in vivo* and *in vitro* results on the biocompatibility of alloys (Čapek et al., 2021). Even current standardized tests (ISO 10993: −5 and −12) cannot mimic the physiological metabolism *in vivo* (Čapek et al., 2021). Therefore, further studies on *in vivo* biocompatibility are greatly warranted.

## 4 Antibacterial Mechanism of Mg-, Zn-, and Fe-Based Alloys as Orthopedic Implants

**FIGURE 1:**
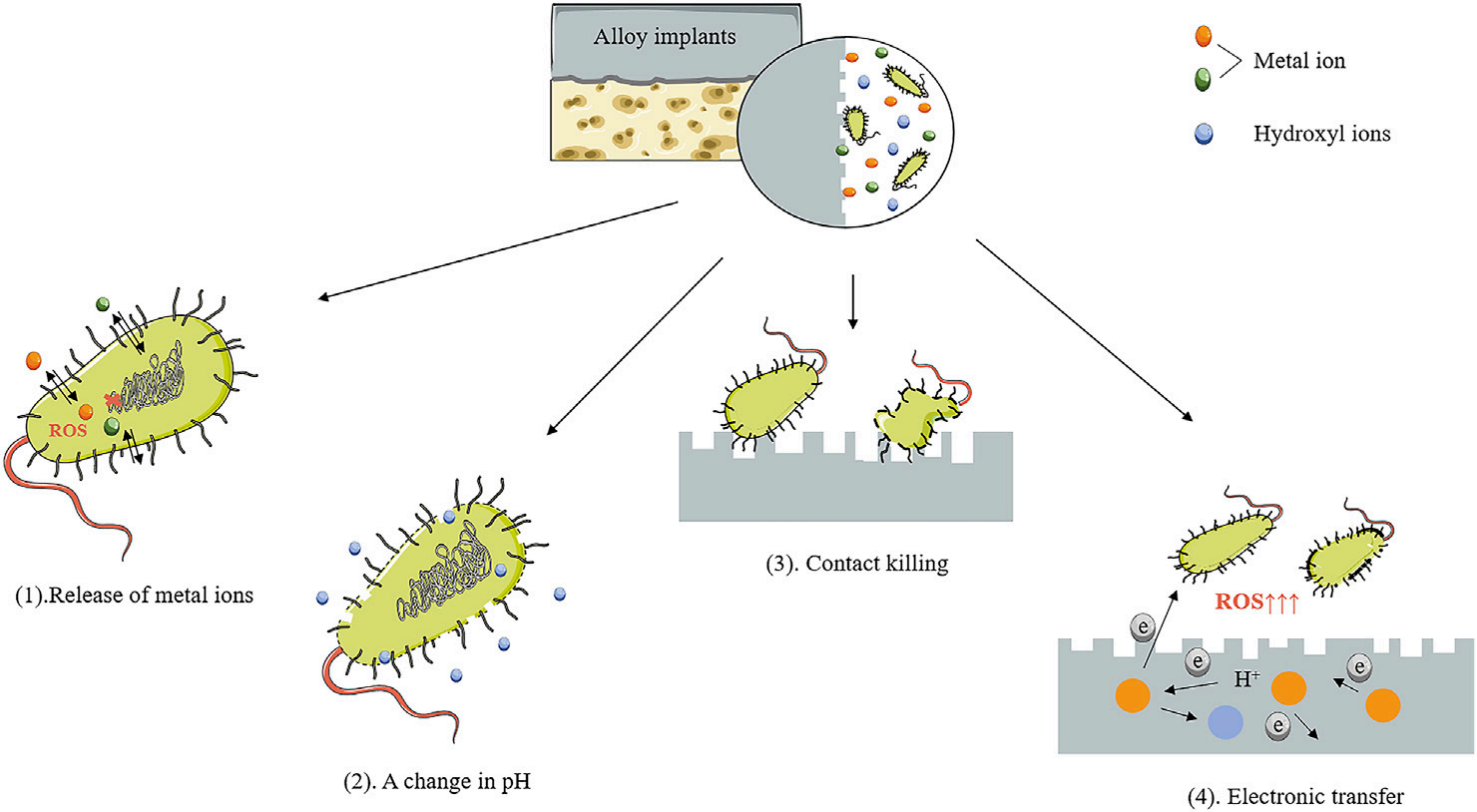
Antibacterial mechanism of Mg-, Zn-, and Fe-based alloys as orthopedic implants. Presently, the proposed antibacterial mechanisms mainly include four aspects: (1) release of metal ions, (2) a change in pH, (3) contact killing, and (4) electronic transfer.

The exact antibacterial mechanisms of alloys have still not been completely elucidated due to their complexity. Nonetheless, a thorough comprehension of antibacterial mechanisms is indispensable for the improvement of the design and application of alloy materials with antibacterial properties as orthopedic implants. Presently, the proposed antibacterial mechanisms mainly include four aspects as follows (Figure 1).

### 4.1 Release of Metal Ions

Metal ions, including Ag^+^, Cu^2+^, and Zn^2+^, released gradually from alloys during the degradation process, are confirmed to inhibit or even kill bacteria *via* multiple pathways. These metal ions are available to bind to bacterial membranes and proteins, thus resulting in greatly increased cell membrane permeability (Feng et al., 2018), which may cause the loss of large volumes of cytoplasm. Simultaneously, these metal ions enter the cytoplasm of bacteria and interact with sulfhydryl groups of proteins, causing irreversible inactivation of proteins owing to the breakage of ionic bonds. These metal ions also produce large quantities of reactive oxygen species (ROS). Ultimately, these events induce the collapse of bacteria’s respiratory and material transport and degradation of DNA (Marambio-Jones and Hoek, 2010; Amin Yavari et al., 2016; Ma et al., 2018). What has been corroborated by *in vitro* studies of Zn-Ag and Mg-Cu alloys is that the expression of genes linked to biofilm formation, bacterial adhesion, autolysis, cell wall biosynthesis, cytotoxicity, and drug resistance is interfered with due to the existence of metal ions (Li et al., 2016b; Li et al., 2018a).

Notably, recent studies have confirmed that Zn^2+^ and Mg^2+^ can also produce immunomodulatory antibacterial activity by influencing the local immune microenvironment. It has been demonstrated that the addition of Zn to hydroxyapatite (HA) can reduce the expression of pro-inflammatory mediator interleukin- (IL-) 8 and the matrix metalloproteinase-9 (Velard et al., 2010). Moreover, under the modulation of the microenvironment created by the implanted scaffolds, macrophages undergo polarization to the anti-inflammatory (M2) phenotype or pro-inflammatory (M1) phenotype and subsequently release a wide series of bioactive molecules, leading to active regeneration of bone or induction of persistent inflammation, respectively. To date, most studies have mainly identified that Zn^2+^ deficiency can aggravate the inflammatory response. Huang et al. demonstrated that Zn^2+^ could promote the polarization of macrophages from M1 to M2 through PI3K/Akt/mTOR pathway. The M2 phenotype polarization of macrophages and the subsequent biological events will create a favorable osteogenic microenvironment (Huang et al., 2021). The immunomodulatory action of Mg^2+^ is related to its concentration. Mg^2+^ at high concentrations has been corroborated to play a role in promoting macrophages polarization to M1 phenotype, increasing the phagocytic ability of the bacteria and expression of TNF-αand iNOS that are crucial to bacterial clearance (Xie et al., 2022). However, persistent and hyperactivated inflammation has an unfavorable effect on bone regeneration (Schmidt-Bleek et al., 2012; Liu et al., 2018a). The corrosion layer produced by the degradation of implants can impede the release of Mg ions (Xie et al., 2022). The environment containing low concentrations of Mg ions are likely to exhibit anti-inflammatory function by suppressing activation of NF-κB, thereby reducing the expression of proinflammatory cytokines in macrophages such as TNF-α, IL-6, and IL-1β (Hu et al., 2018).

**FIGURE 2:**
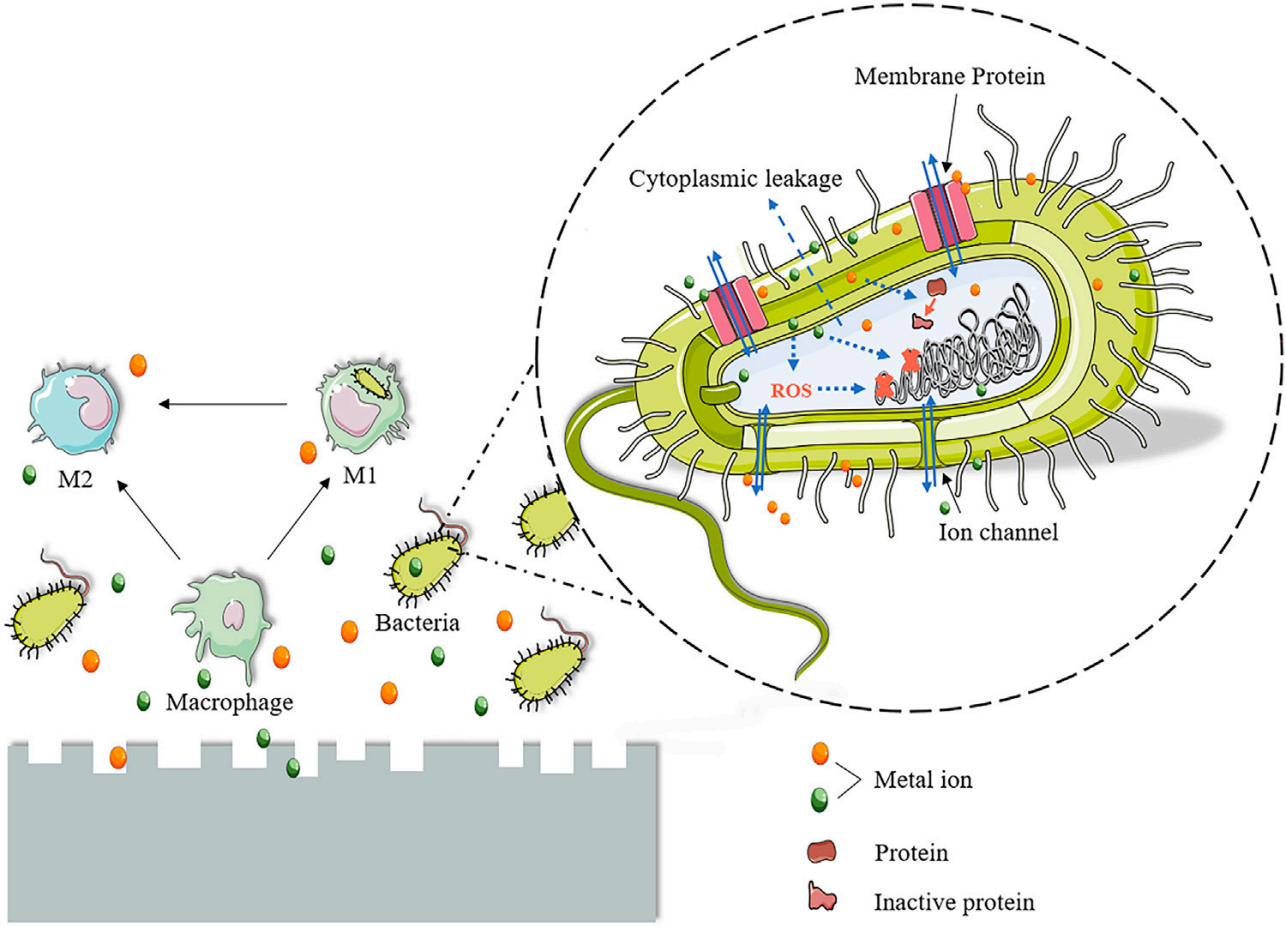
Antibacterial mechanism of metal ions. The effects of metal ions released by alloys on bacteria include two major aspects. On the one hand, these metal ions can bind to bacterial membranes and proteins, resulting in a greatly increased cell membrane permeability, which may lead to massive cytoplasmic loss. At the same time, these metal ions enter the bacterial cytoplasm and interact with the sulfhydryl group of the protein, resulting in irreversible inactivation of the protein due to the cleavage of the ionic bond. Moreover, these metal ions also generate a large amount of reactive oxygen species (ROS). Ultimately, these events lead to the breakdown of bacterial respiration and material transport and the degradation of DNA. On the other hand, metal ions, Zn and Mg, have immunomodulatory antibacterial mechanisms. They can modulate the immune microenvironment by modulating the polarization of macrophages and ultimately play a role in bacterial clearance and killing and the control of inflammation.

Accordingly, the release of metal ions in alloy implants has an integral role in determining the antibacterial activities of alloys (Figure 2). However, it should not be overlooked that although most ions have been confirmed with antibacterial activities that are positively correlated with ions concentration, excessive released ions elicit cytotoxic effects or show safety issues *in vivo*. Therefore, reasonable metal content and metal ion release of the alloys are critically essential.

### 4.2 A Change in pH Value (Creation of an Alkaline Environment)

During the degradation process of Mg-, Zn-, and Fe-based alloys, the pH value of the surrounding environment elevates, accompanied by a massive generation of hydroxyl ions (Li et al., 2019c; Luque-Agudo et al., 2020; Guo et al., 2021c). The pH value appropriate for bacterial survival ranges from 6.0 to 8.0. Both over-acid and over-alkaline environments are detrimental to bacterial growth (Krulwich et al., 2011). The existence of an alkaline environment further enhances the antibacterial property of alloys, which appears prominently in Mg-based alloys. It is regarded as the major antibacterial mechanism of Mg alloys (Qin et al., 2015a; Feng et al., 2018; Luque-Agudo et al., 2020). It has even been demonstrated that the bactericidal effect of Mg is entirely due to the elevation of pH values rather than the release of Mg^2+^ because bacterial growth is not inhibited at all when the pH of the supernatant in the corrosive solution of Mg-based alloys is regulated to neutral or performing bacteriostatic experiment with Mg^2+^ solely (Robinson et al., 2010; Rahim et al., 2015). Besides, the antibacterial property of Mg increases gradually with the augment of pH during the degradation process, suggesting that the antibacterial property of the Mg-based alloys has a positive correlation with the PH value (Robinson et al., 2010; Qin et al., 2015a). According to the *in vitro* experiments by Qin et al. and Rahim et al., when relying solely on high alkaline pH to inhibit bacteria, the pH value of the solution needs to reach 9. When the pH value is greater than 10, it can produce higher antibacterial efficiency. However, the medium with a pH value of 8 cannot produce an antibacterial effect (Qin et al., 2015b; Rahim et al., 2015). In the *in vitro* immersion tests of antibacterial alloys, due to the large differences in the degradation rates of various alloys and the use of different culture solutions, the obtained pH change curves are also different. For some alloys, the pH value of the immersion solution can exceed 9 after immersing for a few hours (Li et al., 2016b; Yan et al., 2019). For example, the pH value of the as-cast Mg-x Cu (x = 0.1, 0.25 wt%) alloy immersion solution exceeded 9 after only 3 h of immersion *in vitro* (Li et al., 2016b). However, the pH value of the immersion solution of some alloys rises slowly, and it takes several days to reach an effective bacteriostatic pH (Qin et al., 2015a; He et al., 2015; Liu et al., 2017b). The zinc-containing Mg-Ca-Sr alloy reported by He et al. did not reach a pH value of 10 until 5 days of immersion (He et al., 2015). In summary, the time an alloy takes to reach an effective bacteriostatic pH ranges from a few hours to a few days, depending on its degradation rate. At the same time, as briefly mentioned above, there may be large differences in the *in vitro* and *in vivo* degradation behavior of alloys. The changes induced by alloy degradation *in vivo* remain to be investigated. Moreover, when Mg-based alloys are used *in vivo*, their antibacterial property will decrease because the high pH value is prone to be gradually buffered by body fluid (Bartsch et al., 2014; Zhao et al., 2020). Bacteria in a high alkalinity environment will release a large amount of H^+^ to partially neutralize the high environmental pH (Qin et al., 2015b). Thus, *in vivo*, the elevation of pH value may only exert bacteriostasis at the early stages (Liu et al., 2016).

### 4.3 Contact Killing (Direct Contact Sterilization)

In addition to fatal killing effects on bacteria caused by metal ions and hydroxyl ions released from alloy degradation, the surface of alloys can also cause damage by direct contact with bacteria. Exposure of bacteria to the surface of alloys leads to the destruction of bacterial membrane and structure, as well as the repression of adhesion (Jiao et al., 2021). Qin et al. confirmed that Zn and Zr on the surface of Mg-Nd-Zn-Zr alloy could suppress the bacterial colonization and exhibited a direct contact killing effect on MRSA (Qin et al., 2015a). The surface of Sr or Ag-doped alloys shows resistance to bacterial adhesion and biofilm formation as well in the experiment of simulating competitive surface colonization by co-culture with cells and bacteria (Cochis et al., 2020). Deng et al. stated that the surface of porous microwave sintered Fe-Cu alloy could cause perforation, deformation, and damage to cell membranes, thus inducing bacterial cell death. Porous structure and Cu-rich precipitation phase play an important role in the mechanical killing process (Deng et al., 2021). The *in vivo* anti-osteomyelitis assays of Mg-Cu alloys showed that bacterial adhesion was not observable on the surface of alloys, which also verified the inhibition of bacterial adhesion on alloy surface (Li et al., 2016b).

### 4.4 Electron Transfer

Electron transfer is essential for bacterial energy metabolism and survival (Gomaa et al., 2022). Electron transfer is an essential link in bacterial respiration (Shi et al., 2016). Disrupting this process will stimulate the massive production of ROS in the bacteria and produce a killing effect on the bacteria (Wang et al., 2018). Metal ions released from alloy implants affect bacterial electron transfer. For example, the interaction between Ag^+^ and bacterial sulfhydryl groups interferes with essential enzymes in the respiratory chain and prevents sufficient electron transfer to oxygen, ultimately leading to the production of large amounts of ROS (Park et al., 2009). In addition, Wang et al. confirmed that active metal ions such as Zn^2+^ and Cu^2+^ might catalyze electron transfer. Moreover, various metal ions can promote each other and coordinately enhance the interference effect on the electron transfer of bacterial ion channels, as suggested by Wang et al. (2016). In their study, the antibacterial effect of multi-component solutions containing Ag^+^, Zn^2+^, and Cu^2+^ was much stronger than that of single-component solutions with the same ionic concentration (Wang et al., 2016), suggesting that multi-component alloys may have better anti-infective properties due to the synergistic antibacterial effect of multiple metal ions. Interestingly, the increase in the pH value caused by alloy degradation also affects the electron transfer of bacteria. As mentioned above, bacteria in a high alkalinity environment produce large amounts of H^+^ to neutralize the high environmental pH value (Qin et al., 2015b). This net transfer of protons to the extracellular compartment disrupts the bacterial transmembrane electrochemical gradient and results in ATP synthesis disorder. Eventually, bacteria die due to abnormal proliferation and metabolism. Furthermore, the potential difference between the components in the alloy implants will lead to electron transfer. Excessive consumption of H^+^ during electron transfer can affect the activity of cellular proton pumps, resulting in a massive release of ROS and the killing of bacteria (Zhang et al., 2021b). Potential differences between the components in alloys result in electron transfer. H^+^ will be consumed in the process of electron transfer, which will affect the activity of the proton pump. This ultimately induces the abundant release of ROS and the killing of bacteria (Lemire et al., 2013).

## 5 Conclusion

It is a feasible and efficient strategy to develop biodegradable alloys with antibacterial properties as orthopedic implants to solve the issues on IAI. Mg-, Zn-, and Fe-based alloys with antibacterial properties hold great application prospects. Nonetheless, studies on Mg-, Zn-, and Fe-based alloys with antibacterial properties as orthopedic implants are still at the exploratory stage. The evaluation of alloy performance is far from perfect, and most alloys still stay at the stage of *in vitro* antibacterial assay. Therefore, *in vivo* evaluation of the alloys needs further refinement to facilitate translation toward clinical application. Furthermore, comparing Mg- and Zn-based alloys, the development of Fe-based alloys with antibacterial properties as orthopedic implants is obviously insufficient. Future studies could devote additional attention to the manufacturing of Fe-based alloys with antibacterial properties. Because the antibacterial mechanisms of alloys remain unclear, studies on mechanisms are still indispensable for further design of alloy implants with antibacterial properties. Overall, there are still some unknowns about biodegradable alloys with antibacterial properties as orthopedic implants, which await exploration in future research, whether from the perspective of design and development or studies on antibacterial mechanisms.
